# Vacancy-Engineered Phonon Polaritons in a van der Waals Crystal

**DOI:** 10.1021/acsnano.5c20443

**Published:** 2026-05-15

**Authors:** Mashnoon Alam Sakib, Naveed Hussain, Mariia Stepanova, William Harris, Joshua J. Bocanegra, Juan Diego Sanchez, Camilo Velez, Ruqian Wu, H. Kumar Wickramasinghe, Maxim R. Shcherbakov

**Affiliations:** † Department of Electrical Engineering and Computer Science, University of California, Irvine, California 92697, United States; ‡ Department of Physics and Astronomy, University of California, Irvine, California 92697, United States; § Department of Mechanical and Aerospace Engineering, University of California, Irvine, California 92697, United States

**Keywords:** α-molybdenum trioxide, phonon-polaritons, nanophotonics, oxygen vacancy defects, stoichiometry, density functional theory, photoinduced force microscopy

## Abstract

Phonon polaritons (PhPs) in low-symmetry van der Waals (vdW) materials enable deep-subwavelength control of mid-infrared light for nanoscale optics and sensing. However, intrinsically reconfiguring their dispersion without external fields, lithography, or chemical intercalation has remained elusive. Here, we introduce a thermomechanical approach that tunes PhPs in α-molybdenum trioxide (α-MoO_3_) through controlled oxygen vacancy formation and lattice strain. Near-field nanoimaging reveals an average polariton wavevector shift of Δ*k*/*k* ≈ 0.13 within the lower Reststrahlen band. Stoichiometric analysis, density functional theory, and finite-difference time-domain simulations indicate vacancy concentrations of 1–2% and ≈–1.2% compressive strain, resulting in a dielectric permittivity modulation of up to ≈15%. Despite these structural perturbations, polariton lifetimes remain high (1.15 ± 0.29 ps). This work offers thermomechanical vacancy engineering as a robust route for reprogrammable polaritonic response in vdW crystals for nonvolatile nanophotonic architectures.

## Graphical abstract

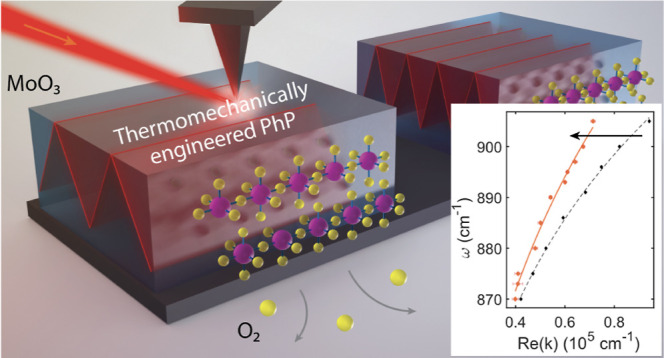

## Introduction

Phonon polaritons (PhPs)—quasiparticles arising from the coupling of light to lattice vibrations—offer a compelling route to manipulate electromagnetic fields at the nanoscale.^1–4^ In van der Waals (vdW) crystals, PhPs can confine free-space mid-infrared (MIR) light deep below the diffraction limit,^5–9^ offering applications in subdiffraction imaging,^8,10^ long-range hyperlensing,^11,12^ and engineered photonic states of matter.^7,13,14^ The key challenge, however, remains in the control of PhP dispersion through material engineering. While substantial efforts have been made to manipulate PhPs across various engineered and natural material platforms, including by altering the dielectric environment^15–19^ incorporating phase change materials,^20–22^ interface-mismatched strain-engineering,^16^ and by suspending polar materials in air;^14,23,24^ a robust stoichiometry-based method for reconfiguring PhPs in vdW crystals remains elusive.

α-Phase molybdenum trioxide (α-MoO_3_) has emerged as an ideal platform for intrinsically reconfiguring phonon polaritons via lattice engineering. As a low-symmetry, 2D-layered wide-bandgap (*E*_g_ = 2.93 eV) vdW transition metal oxide supporting low-loss in-plane hyperbolic PhPs,^4,7,25^ α-MoO_3_ exhibits extreme sensitivity to physical and chemical modifications, allowing the exploitation of natural stoichiometry as an alternative dispersion tuning pathway. Introducing intercalants, such as hydrogen,^26^ tin and cobalt,^27,28^ and isotope enrichment^9,29^ can substantially modify the PhP dispersion; however, the introduction of foreign atomic species may lead to additional perturbation of the intrinsic homogeneity of the crystal lattice that could adversely impact the polariton lifetimes.^1,4,26–30^ In contrast, oxygen vacancies (OVs)—naturally occurring and controllable in α-MoO_3_—offer an attractive avenue to engineer its stoichiometry.^31,32^ The stoichiometry of α-MoO_3_ spans from a wide-bandgap oxide with abundant Mo^6+^, to intermediate reduced oxides (MoO_3–*x*_, 0 < *x* < 1), to semimetallic MoO_2_ with a reduced oxidation of Mo^4+^.^33,34^ Controlling oxygen content may lead to tunable intrinsic mass composition, allowing targeted modification of optical and acoustic phonons^26^ and modulation of the local dielectric permittivity ε, thereby offering a route to on-demand control of PhP dispersion.

In this work, we demonstrate that thermomechanically induced OVs and compressive strain enable tunable PhPs in α-MoO_3_. By hot-pressing α-MoO_3_ flakes in a pressure- and temperature-controlled environment, we selectively extract the loosely attached oxygen atoms near the vdW gaps and induce nonvolatile thermal expansion-mismatch-driven strain.^36^ Near-field nanoimaging at room temperature with photoinduced force microscopy (PiFM)^37^ reveals an average polariton wavevector shift of Δ*k*/*k* ≈ 0.13 within the lower Reststrahlen band (L-RB) for processing temperatures between 160 and 200 °C. The presence of OVs and strain is confirmed by Raman spectroscopy,^35^ X-ray photoelectron spectroscopy (XPS), and grazing-incidence X-ray diffraction (GIXRD) measurements. Density functional theory (DFT) and numerical finite-difference time-domain (FDTD) calculations show agreement with experimental results and suggest oxygen vacancies with concentrations of 1%–2% along with a –(1.2 ± 0.2)% compressive strain, yielding a stable dielectric permittivity modulation of up to Δε/ε ≈ 0.15. The lifetimes of tuned phonon-polaritons remain high at 1.15 ± 0.29 ps with an average lifetime loss of only 29% compared to the pristine material. Our findings establish a previously unexplored tuning mechanism for MIR polaritons, offering a route for reprogramming of light-matter interactions in vdW materials.

## Results and Discussion

### Nanoimaging PhPs in Hot-Pressed α-MoO_3_

Figure 1a shows a schematic representation of the thermomechanical processing of α-MoO_3_ flakes. Pristine α-MoO_3_ flakes are mechanically exfoliated onto a polished silicon substrate, followed by capping with an identical silicon substrate; see Methods and Experimental Procedures. This capped assembly is put inside a pressure device assembly (PDA). The PDA consists of steel bars that generate a localized uniaxial pressure via the top substrate by tightening the screws on the sides of the PDA. The uniaxial pressure exerted by the PDA is found crucial for cutting off oxygen supply during heating of the assembly in a muffle furnace up to temperatures ranging from 50 to 400 °C. Thermomechanical hot-pressing also ensures interfacial adhesion with the Si substrate during the relative thermal expansion and contraction processes, offering maximum strain transfer.^36,38,39^ To analyze how the processing induces changes on PhP propagation dynamics and modify their fundamental characteristics, we use PiFM experiments for nanoimaging PhP propagation on pristine and hot-pressed flakes. Figure 1b illustrates the PiFM technique. Here, a Pt/Ir-coated tip is obliquely illuminated with MIR light to excite tip-launched PhPs. PiFM records the *z*-component of the local field distribution by mechanically detecting the optical force acting on the tip.^25,40,41^ We record the PhP propagation in pristine and hot-pressed α-MoO_3_ flakes within the L-RBs spanning 865–915 cm^–1^ along the [100] direction. In Figure 1c,d, we show PiFM images taken at 900 cm^–1^ for PhPs in pristine and hot-pressed (at 185 °C) flakes of similar thicknesses, respectively. The scale bars are 2 μm. The PiFM signal, outlined by the dashed blue line in Figure 1c and corresponding to a typical cross-section of the pristine flake with thickness *t*_pr_ = 107 ± 2 nm, reveals PhPs with a wavelength of λ_pristine_^PhP^ = 816 nm and a propagation length of *L*_pr_ = 1.17 ± 0.32 μm (see Supporting Information Figure S1 and S2). In comparison, the hot-pressed flake, showing a typical PiFM signal cross-section with a dashed red line in Figure 1d with a thickness of *t*_hp_ = 102 ± 2 nm, reveals an elongated PhP wavelength of λ_hot‑pressed_^PhP^ = 892 nm and a propagation length of *L*_hp_ = 1.45 ± 0.38 μm. In Figure 1e, we compare the PhP profiles for the pristine and 185 °C hot-pressed flakes recorded from the blue and red lines in Figure 1c, respectively. At the same excitation frequency of 900 cm^–1^, the 185 °C hot-pressed flake, despite having a 5 nm lower thickness, shows an approximately 10% increase in PhP wavelength. It is worth noting that the PhP wavevector is typically inversely proportional to the thickness of the flakes, shifting the PhP dispersion for thinner flakes to the higher-momentum region.^1,6,7^ In order to record the dispersion and analyze this PhP propagation characteristic across the L-RB of α-MoO_3_, we recorded PhP signals spanning from 865 to 915 cm^–1^; see Supporting Information Figure S1 and S2. We found the PhP wavelength for processed flakes is larger across all the measured frequencies compared to those of pristine flakes. We plot the extracted PhP wavevector against the excitation frequencies to map the dispersion characteristics in Figure 1f for the pristine (blue) and 185 °C hot-pressed (red) flakes. Figure 1f suggests that thermomechanical processing of the flakes at 185 °C shows an average 7.6% dispersion shift compared to the pristine flake, despite the latter being 5 nm thicker.

**Figure 1:**
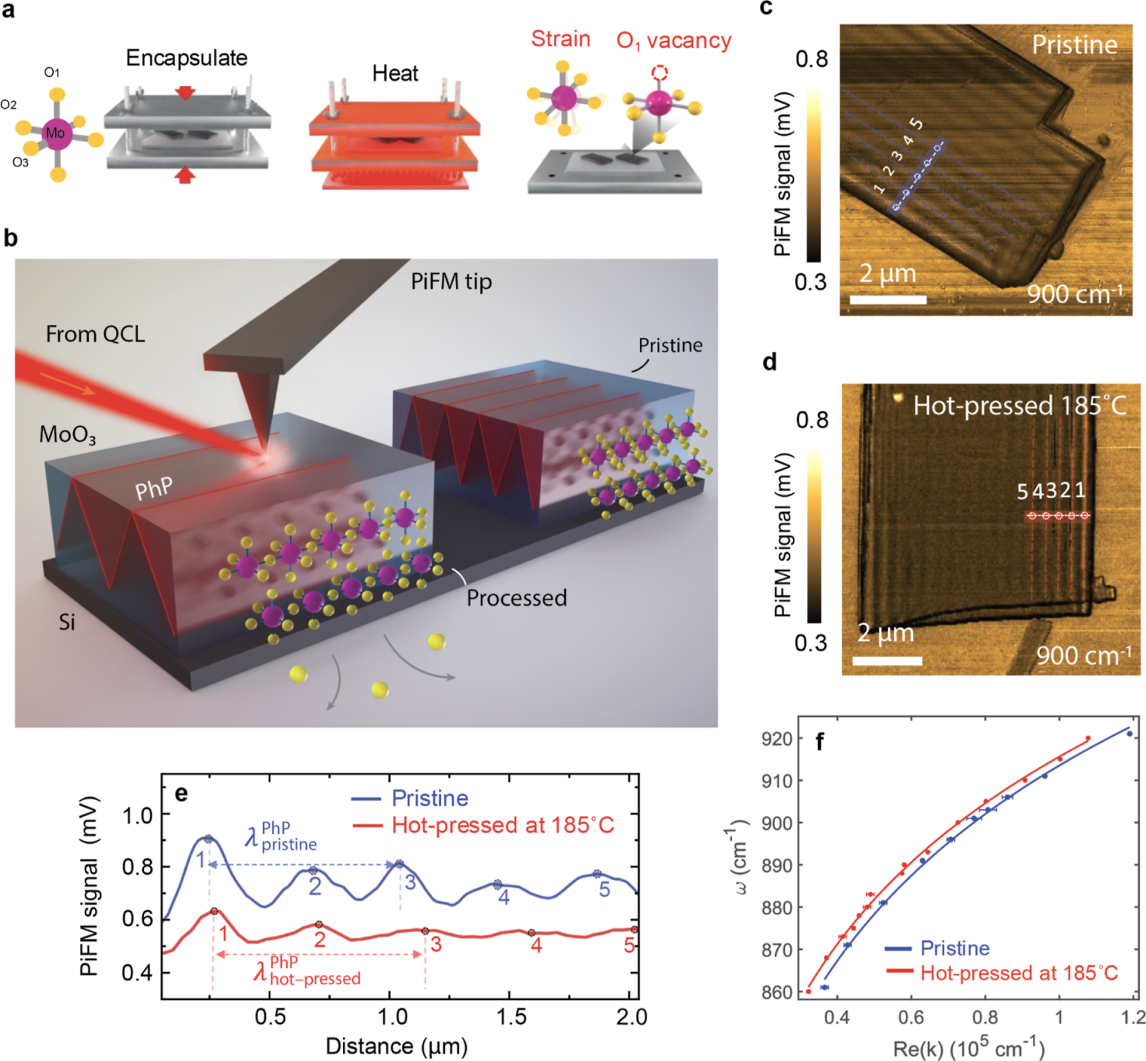
Thermomechanical hot pressing induces strain and oxygen vacancy defects in α-MoO_3_ and enables MIR PhP dispersion engineering. (a,b) Schematic illustrations of (a) thermomechanical processing and (b) photoinduced force microscopy (PiFM) measurements. (c,d) PiFM images measured at 900 cm^–1^ for a pristine flake (c, *t*_pr_ = 107 ± 2 nm) and a flake hot-pressed at 185 °C (d, *t*_hp_ = 102 ± 2 nm). The scale bars are 2 μm. (e) PiFM map cross-sections showing λ^PhP^ increase in the hot-pressed flake. (f) PhP dispersion *k*(ω) across the L-RB (ω = 865–915 cm^–1^); solid lines: polynomial fits.

### PiFM Characterization of PhP Dispersion Modulation

We use PiFM to visualize propagation of PhPs in pristine and hot-pressed α-MoO_3_ flakes processed at 160 °C, 185 °C and 200 °C; the full data sets for these flakes are given in Supporting Information Figures S1–S3, respectively. Figure 2 summarizes these data sets. Figure 2a,b shows schematic illustrations of (a) PiFM tip-launched PhPs and (b) FDTD method with a point-like electric-dipole radiation source launched PhPs in pristine α-MoO_3_ membrane exfoliated on a Si substrate; see Methods and Experimental Procedures and Supporting Information Figures S5–S7. Figure 2c shows the PiFM-recorded PhP dispersion within the L-RB, recorded from a pristine α-MoO_3_ flake with thickness, *t*_pr_ = 107 ± 2 nm (solid blue curve). To build a reference model for the quantitative analysis of PhPs, we performed FDTD calculations to simulate α-MoO_3_ PhPs for frequencies spanning the L-RB. The dispersion calculated with FDTD for *t*_pr_ = 107 nm flake is shown in Figure 2c in black. Figure 2c suggests an agreement between PiFM and FDTD, with an average error of 1.6% between them, mostly originating from the high-frequency area; we attribute the errors to having arisen from the approximate character of the substitution of a real tip with an extended dipole in this approach. This result suggests that the pristine model is a good reference to compare the results obtained with PiFM to the PhP dispersion expected in the pristine flake of the same thickness. This way, any systematic deviations between the PiFM and the FDTD data would point to a tunable response in processed flakes. Moreover, because hot-pressing inevitably alters the thickness of MoO_3_ flakes, a direct before-and-after comparison on the same flake could conflate the thickness-dependent phonon-polaritonic effects compared to those induced by oxygen vacancy- and strain-formation. Thus, to keep our primary focus on the vacancy-engineered modulation of PhPs, we recorded and compared near-field data among different flakes with closely matched thicknesses—rather than on the same flake before and after processing. This ensures that the observed changes primarily reflect oxygen-vacancy formation rather than thickness variations, and thus enables a more reliable assessment that reveals the isolated effects arising from oxygen-vacancy and strain-driven PhP modulation.

**Figure 2:**
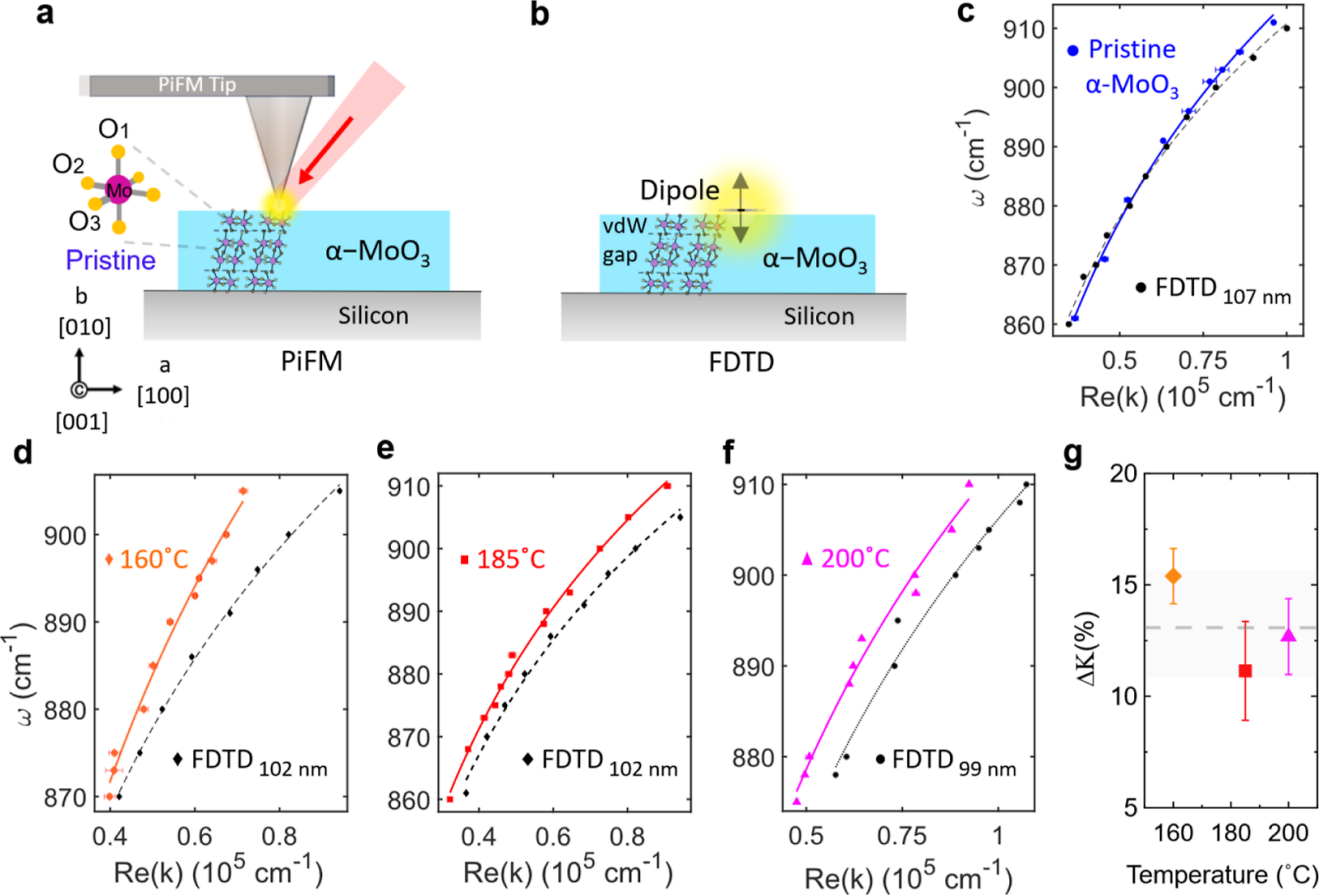
PiFM and FDTD characterization of PhPs propagation for pristine and hot-pressed α-MoO_3_ flakes. (a,b) Illustrations of (a) PiFM tip-launched PhPs and (b) FDTD-modeled PhPs with a point-like electric-dipole radiation source in a α-MoO_3_ on Si. (c) PiFM-recorded dispersion from a pristine 107 nm-thick α-MoO_3_ flake (blue) is plotted against FDTD-simulated dispersion from a flake of the same thickness (black). (d–f) PhP dispersion plotted for processed α-MoO_3_ flakes with temperatures at (d) 160 °C, (e) 185 °C, and (f) 200 °C with flake thicknesses of 102 ± 2 nm, 102 ± 2 nm and 99 ± 2.5 nm, respectively. Corresponding FDTD-simulated dispersion from pristine α-MoO_3_ flakes with the same thicknesses are plotted with dashed black curves for comparison. (g) Thermomechanical dispersion modulation extracted from the corresponding hot-pressed α-MoO_3_ flakes processed at 160 °C, 185 °C and 200 °C. The error bars represent fitting errors.

We perform PiFM experiments for hot-pressed α-MoO_3_ flakes at 160 °C, 185 °C and 200 °C and show their dispersion relationships in Figure 2d–f. The flake thicknesses for these cases are measured as *t*_160 °C_ = *t*_185 °C_ = 102 ± 2 nm and *t*_200 °C_ = 99 ± 2.5 nm; for topography data, see Supporting Information Figure S3. To analyze and compare the dispersion relationships, we use FDTD calculations on a pristine α-MoO_3_ flake and plot it in Figure 2d,e (with *t*_160 °C_ = *t*_185 °C_ = 102 nm) and in Figure 2f (with *t*_200 °C_ = 99 nm) against the PiFM data. It can be seen that hot pressing modulates PhP dispersion toward the low-momentum region by an average 15.4%, 11.2% and 12.7% at 160 °C, 185 °C and 200 °C, respectively, with an average 13.1% dispersion modulation, as shown in Figure 2g. This capability to modify the PhP wavelength presents the potential for achieving on-demand dispersion configurability in vdW materials.^7,13,14^ The highest wavelength elongation for L-RBs in hot-pressed α-MoO_3_ is observed to be up to 24%. We attribute this elongation to OV and strain-induced nonvolatile increase in the dielectric permittivity for hot-pressed α-MoO_3_, as discussed below. To characterize the PhP propagation dynamics, we extract the polariton lifetimes, τ, by fitting the PhP linescans to an exponentially decaying sinusoidal function; see Supporting Information Figures S1–S3. The calculated polariton lifetimes in thermomechanically processed α-MoO_3_ flakes are 1 ± 0.2 ps, 1.4 ± 0.2 ps and 1.1 ± 0.3 ps for 160 °C, 185 °C and 200 °C, respectively. These lifetimes are comparable to those in pristine α-MoO_3_ flakes, demonstrating the resilience of PhP propagation after the introduction of OVs and strain into the α-MoO_3_ crystal homogeneously across the L-RB band. For lifetimes measured at L-RB frequencies spanning from 871 to 915 cm^–1^, the PhP propagation characterization suggests a reasonable average 29% loss, establishing hot-pressing as a robust method to tune the PhP propagation when compared to other reported works that involve modification of the homogeneity of the crystal.^9,26,28,29^

### Stoichiometry Characterization of Hot-Pressed α-MoO_3_

To establish the nature of the observed PhP tunability, we extensively characterize pristine and hot-pressed flakes using Raman spectroscopy, X-ray photoelectron spectroscopy (XPS) and grazing-incidence X-ray diffraction (GIXRD) allowing us to identify OVs and strain as the primary reasons for dielectric permittivity modulation.

Figure 3a shows the Raman spectra of pristine and hot-pressed flakes in a muffle furnace under ambient conditions at 50, 100, 150, 250, 350, and 400 °C. The spectrum revealed vibrational bands that correspond to the stretching modes between 1000 and 600 cm^–1^, deformation modes between 600 and 400 cm^–1^, and lattice modes below 200 cm^–1^. All samples displayed distinct and robust bands at 117, 129, 245, 284, 291, 338, 665, 819, and 996 cm^–1^. A typical α-MoO_3_ crystal comprises a double layer of linked and deformed MoO_6_ octahedra and is thermodynamically stable.^42,43^ The relatively weak peak at 996 cm^–1^ is the A_g_ mode attributed to the asymmetric Mo^6+^=O­(1) stretching mode of terminal oxygen along the *b*-axis. The band at 819 cm^–1^ is the most intense band that corresponds to the symmetric stretching mode of doubly coordinated oxygen Mo^2^=O­(3), which originates from the oxygen shared by the two MoO_6_ octahedra and is sensitive to oxygen vacancies and defects.^44^ The full width at half-maximum (fwhm) of this peak provides crucial information regarding the presence of OVs in α-MoO_3_ flakes. The peak broadens as hot-pressing temperature increases, with the fwhm increasing from 8.2 cm^–1^ (for pristine) to 15.12 cm^–1^ (for flakes h.p. at 350 °C); see Supporting Information Figure S8. This broadening reflects a reduced phonon lifetime due to increased local disorder associated with oxygen vacancy formation. In Figure 3b illustrates an oxygen vacancy defect (OVD) at the O_1_ site of h.p. α-MoO_3_. The presence of OVs can be assessed by changes in the ratio of Raman mode intensities of B_2g_ at 284 cm^–1^, and B_3g_ at 291 cm^–1^ as B_2g_/B_3g_.^45–47^ In Figure 3c, we show the variation in the ratio of Raman mode intensities B_2g_ and B_3g_ from hot-pressed α-MoO_3_ flakes processed with temperatures from 150 to 200 °C with steps of 10 °C. Here, for pristine α-MoO_3_, the intensity of the B_3g_ shoulder peak corresponds to an intrinsically small amount of oxygen vacancies. We observed a consistent increase in this peak’s intensity, making it the primary marker of OV creation. See Supporting Information Figure S9 for the calculated ratio of Raman mode intensities for hot-pressed α-MoO_3_ flakes from 50 to 350 °C.

**Figure 3:**
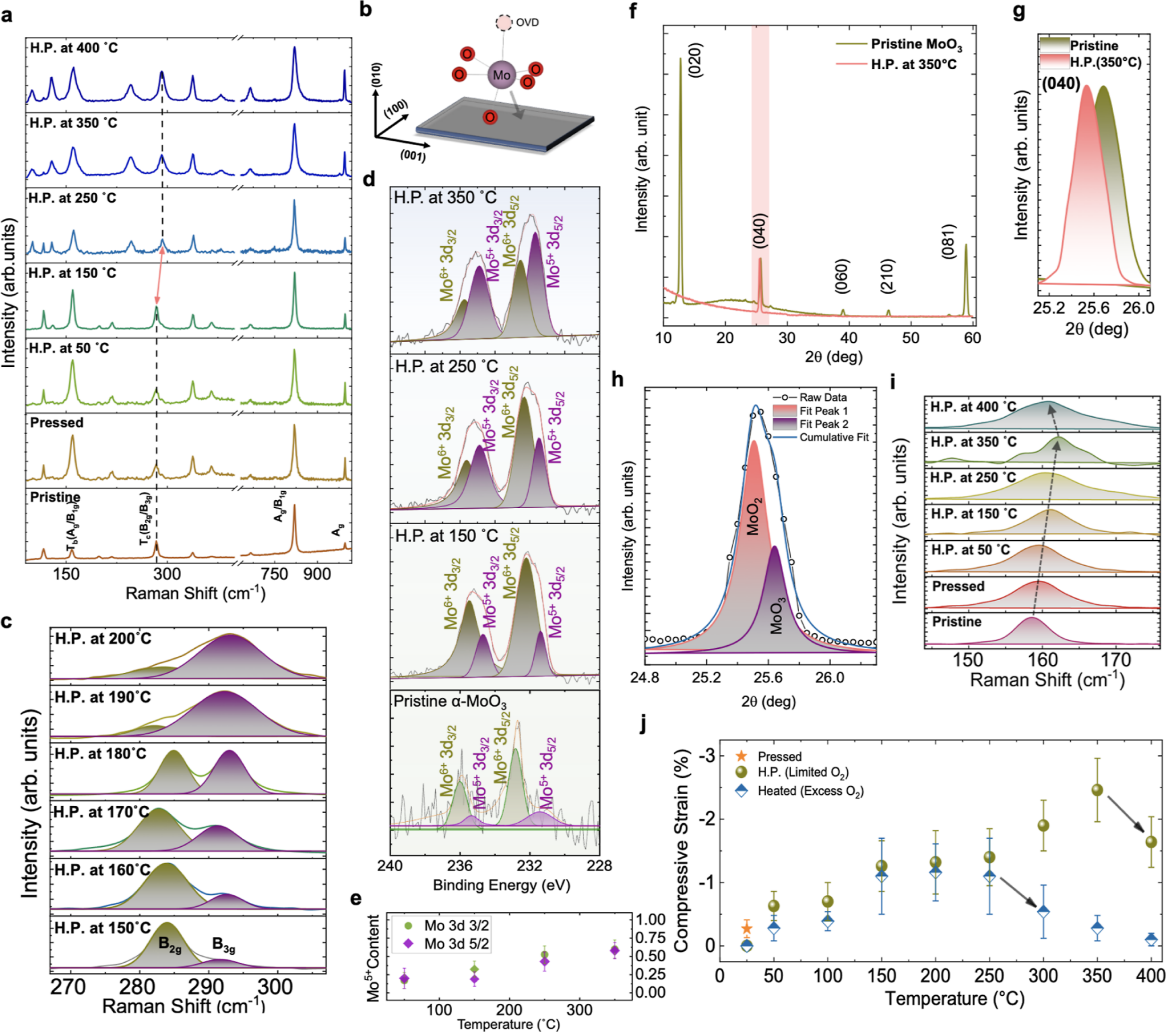
Stoichiometry characterization of hot-pressed α-MoO_3_. (a) Raman spectra of the pristine, pressed and h.p. α-MoO_3_ with T_b_ and T_c_ mode highlighted around 150 cm^–1^ and 290 cm^–1^ with shaded regions, respectively. (b) Illustration of an oxygen vacancy (OV) defect at the O_1_ site of h.p. α-MoO_3_. (c) Magnified Raman spectra (normalized) of T_c_ (B_2g_/B_3g_) mode acquired from h.p. α-MoO_3_ from 150 to 200 °C show a band transition with a decrease in oxygen-to-metal ratio, highlighted by orange arrow in (a). (d) Mo 3d scan of pristine and h.p. flakes obtained from XPS to probe the processed α-MoO_3_ stoichiometry. (e) Mo^5+^ content in α-MoO_3_, calculated using peak area fitting of Mo 3d_3/2_ and 3d_5/2_ peaks. (f) GIXRD pattern of the pristine and h.p. flakes processed at 350 °C. (g) Comparison of the (040) diffraction peak of pristine and h.p. α-MoO_3_ at 350 °C, showing a shift by 0.4°. (h) Deconvolution of (040) diffraction peak of h.p. flake yields a ratio of MoO_2_ and MoO_3_ of 6:4. (i) T_c_ shifts suggest –(1.2 ± 0.2)% compressive strain in flakes processed at 150–200 °C. (j) Comparison of compressive strain induction in heated and thermomechanically processed h.p. flakes as a function of processing temperature.

To further assess the presence of the introduced OVs, we performed XPS to identify the stoichiometry of pristine and h.p. α-MoO_3_. In Figure 3d, we show the Mo 3d scans of pristine, h.p. at 150, and 350 °C samples. For pristine α-MoO_3_, Mo 3d_3/2_ and Mo 3d_5/2_ are located at binding energies of 235.92 and 232.82 eV, respectively, which corresponds to pure α-MoO_3_ stoichiometry with Mo^6+^ oxidation state with hexavalent formal molybdenum ions.^48,49^ As the temperature rose to 350 °C, the h.p. flakes demonstrated a gradual rise in the proportion of the Mo^5+^ state (purple peaks), indicating a shift in the composition toward a lower oxidation state (MoO_3–*x*_). The calculations based on fitted peak areas revealed that the h.p. flakes represented a hybrid system, with the Mo^5+^ content in MoO_3–*x*_ increasing from 18.7% at 25 °C to approximately 58.8% for the flakes hot-pressed at 350 °C, as shown in Figure 3d,e. XPS results complement the Raman results and suggest that the chemical stoichiometry can be controlled by the introduction of OVs with increase in h.p. temperature. The XPS survey spectrum of hot pressed α-MoO_3_ on gold coated Si substrate and XPS-mapped reduced oxidation state (extracted from spectral peak fitting) are shown in Supporting Information Figure S9–S11. Additionally, GIXRD results shown in Figure 3f–h indicate the transition from polycrystalline to monocrystalline phase in h.p. flakes as there were no peaks detected other than (040) along the basal plane at 25.53°, compared to its pristine counterpart. However, in Figure 3g, we observe a shift of 0.4° in (040) peak, which indicates the existence of MoO_3–*x*_.^50,51^ Gaussian peak fitting suggested that the MoO_2_/MoO_3_ ratio in a 350 °C h.p. flake was 6:4 (Figure 3h). XPS results indicate a partial reduction of α-MoO_3_ (Mo^6+^) to α-MoO_3–*x*_ (Mo^5+^) as well.

In addition to OVs, to quantify the lattice crystal strain in the hot-pressed α-MoO_3_ flakes, we probed the changes in *T*_b_ (A_g_/B_1g_) mode, the translational chain Raman mode at 158.4 cm^–1^ which is critical in determining the lattice strain in α-MoO_3_. In Figure 3i,j, we estimate the lattice strain for h.p. flakes. Figure 3i shows a consistent blue shift in T_b_ mode originally located at 158.4 cm^–1^, for samples hot-pressed up to 350 °C before relaxing back to its position at 400 °C. The amount of strain and its type were calculated as $\delta \left(\%\right)=\left(\omega_{T_{b}}^{pristine}-\omega_{T_{b}}^{h.p.}\right)/\omega_{T_{b}}^{pristine}\times 100$ where $\omega_{T_{b}}^{pristine}$ and $\omega_{T_{b}}^{h.p.}$ are the frequencies of the T_b_ phonon mode corresponding to pristine and hot-pressed α-MoO_3_, respectively. We attribute the *T*_b_ mode shift to the compressive strain caused by the coefficient of thermal expansion (CTE) mismatch at the interface between silicon substrate and α-MoO_3_,^36^ while the relaxation is caused by the slippage that supersedes the interfacial adhesion above 350 °C. Because the substrate transfers compressive strain, boosting interlayer adhesion between the silicon substrate and α-MoO_3_ flakes owing to uniaxial pressure; pressure from the top substrate is critical. The applied pressure does not directly generate strain in the lattice; rather, it suppresses interfacial slippage and enables efficient transfer of in-plane thermomechanical strain arising from the CTE mismatch between α-MoO_3_ and the Si substrate during the heating–cooling cycle. To study the impact of uniaxial pressure on compressive strain, we additionally examined heated samples without any uniaxial pressure from the upper substrate. Supporting Information Figure S3b shows the estimated compressive strain values for hot-pressed and bare heated samples. Figure 3j shows that in bare heated samples, the lack of uniaxial pressure results in interfacial slippage at approximately 250 °C, which restricted the maximum compressive strain transfer to –(1.2 ± 0.2)%. In contrast, interfacial slippage in hot-pressed samples was observed at elevated temperatures (350 °C), resulting in a 2-fold increase in the maximum compressive strain transfer –(2.4 ± 0.2)%. We attribute the residual compressive strain to thermomechanical strain transfer arising from the CTE mismatch between α-MoO_3_ and Si under strong interfacial adhesion. Because this process is not purely elastic and reversible, a residual lattice distortion remains after thermal relaxation. Comparable Raman shifts in capped and open heating configurations indicate that strain retention does not require oxygen-vacancy formation.

During thermomechanical processing, the α-MoO_3_ flakes are heated to temperatures at which partial thermal reduction of MoO_3_ is known to occur, leading to the formation of oxygen vacancies.^52^ At elevated temperatures, oxygen can leave the lattice via thermally activated diffusion. Importantly, this process is not thermodynamically reversible during thermal relaxation under our experimental conditions. Reincorporation of oxygen into the lattice would require sufficiently high external oxygen chemical potential to drive reoxidation of MoO_3–*x*_ back to stoichiometric MoO_3_. Under the processing conditions used here, such conditions are not present; therefore, oxygen that leaves the lattice during heating is not reintroduced during cooling. As a result, the material remains in a substoichiometric MoO_3–*x*_ state after the thermal cycle, consistent with previous studies showing that oxygen-deficient molybdenum oxides can remain stable under ambient conditions and reoxidize only under sufficiently oxygen-rich environments or elevated temperatures.^52^

To summarize, the stoichiometric analysis of the hot-pressed α-MoO_3_ confirms the controllable creation of OVs and compressive strain in a wide range of processing temperatures, highlighting their potential roles in the observed PhP modulation.

### DFT Calculations of Strain- and OV-Induced Index Modulation

To explain the dispersion-modulated PhP wavelength elongation, we consider both thermomechanically induced strain and OVs as the primary causes, as evidenced by the stoichiometric analysis. We employ density functional theory (DFT) and FDTD methods to analyze and compare the synergistic effects of strain- and OV-induced index modulation across various OV concentrations and crystal strain levels. The results suggest that the elongation of λ^PhP^ can be attributed to an increase in the static dielectric constants of hot-pressed α-MoO_3_, driven by both OVs and lattice strain. The static dielectric shifts are assumed to combine linearly under small material modulations, such that Δε = Δε_OV_ + Δε_Strain_.

DFT calculations estimate the static dielectric constants of α-MoO_3_ with various defect concentrations utilized with a 3 × 3 × 1 supercell, with oxygen vacancies positioned near the vdW gap as shown in Figure 4a, as these sites are the most energetically shallow. For the details of DFT calculations, see Methods and Experimental Procedures. Up to three defects per supercell were considered. While the relative spatial position of the defects does not change the direction of the dielectric trend, it does affect the magnitude of the shifts; see Supporting Information Figure S12. This site-specific variation is expected to average out under typical experimental conditions. Moreover, as shown in Figure 4c, the results indicate an increase in the dielectric response with an approximately linear trend. A dielectric permittivity modulation of up to ∼8% is observed for up to ∼3% oxygen vacancy concentration. We also found strain to influence the dielectric properties, particularly along the [100] direction. This dependence is attributed to the strong modulation of the conduction band near the gamma point from [100] strain, which, in turn, modulates the band gap; see Supporting Information Figure S13. At these small OV concentrations and strain magnitudes, both mechanisms modify the lattice polarizability independently, allowing their contributions to combine approximately linearly. Moreover, we recorded the room temperature current–voltage (*I*–*V*) characteristics of pristine and hot-pressed α-MoO_3–*x*_-based devices (See Supporting Information Figure S14). Both pristine and hot-pressed samples exhibit nonlinear Schottky-like *I*–*V* behavior and upon fitting both the forward-bias regime with a standard Schottky diode model show that hot-pressed α-MoO_3–*x*_ show a 50% lower resistance compared to pristine α-MoO_3_ indicating an increase in charge carrier concentration by a factor of 2. The incremental charge carrier increase, along with the persistence of *I*–*V* nonlinearity and Ohmic regime absence, suggest that hot-pressing is transitioning from a diffusion-dominated transport in pristine α-MoO_3_ to recombination-dominated transport in substoichiometric α-MoO_3–*x*_. This is consistent with OV-mediated defect assisted conduction. We calculated the Drude-modified dielectric permittivity contributions caused by in charge carriers (see Supporting Information Figure S15). The Drude-modified dielectric permittivity model shows that the surplus of free carriers is insufficient to cause meaningful plasma hybridization of PhPs in hot-pressed α-MoO_3–*x*_.

**Figure 4:**
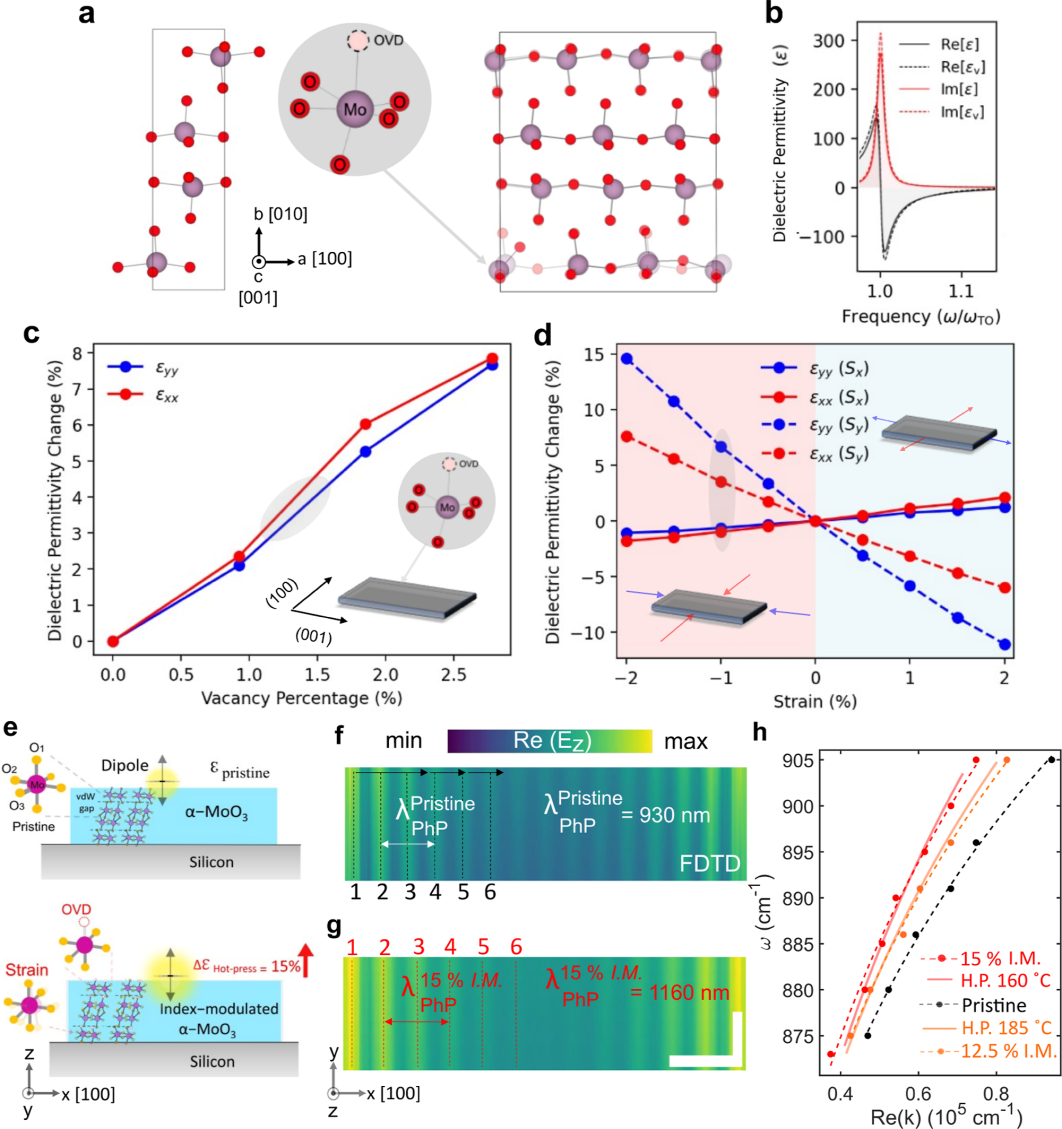
DFT and FDTD analysis of dielectric permittivity modulation in hot-pressed α-MoO_3_. (a) Crystal structure of relaxed pristine and processed α-MoO_3_ with an oxygen vacancy at the O_1_ site near vdW gap. (b) IR dielectric response along the [100] direction of pristine (solid lines) and oxygen deficient (dashed lines) α-MoO_3_. ω_TO_ is the transverse optical phonon frequency. (c,d) Static dielectric constants as functions of (c) OV concentration near the vdW gap, and (d) applied strain along the *x* [100] and *y* [001] directions, including both compressive and tensile strain. DFT calculations of OV-induced α-MoO_3_ suggest dielectric changes of up to 8% with OV concentrations reaching up to 3%. (e) Illustration of pristine and hot-pressed permittivity modeling using FDTD. (f,g) Numerically simulated field distributions Re­(*E*_*z*_) along the *X*–*Y* plane for the pristine (f) and for the index-modulated α-MoO_3_ (g). The scale bar along *X*(*Y*) is 1 μm (0.1 μm). (h) Numerically calculated dispersion of PhPs for a 102 nm α-MoO_3_ membrane for pristine and for permittivity modulation of 12.5% and 15%.

We use FDTD to simulate the effects of increased permittivity on PhP in hot-pressed α-MoO_3_. Here, we model the OV and strain-induced index modulation by modifying the intrinsic static dielectric constants of α-MoO_3_. We illustrate pristine and OV and strain induced permittivity modeling using FDTD in the schematic in Figure 4e. We run FDTD with 102 nm-thick α-MoO_3_ flakes with both the pristine and the 15% index-modulated α-MoO_3_ model; see Supporting Information Figure S5. We show the simulated real part of the out-of-plane component of the electric field (Re­(*E*_*z*_)) along the *X*–*Z* plane for the pristine and for OV-populated and strained α-MoO_3_ in Figure 4f,g, respectively. Simulated Re­(*E*_*z*_) shown in Figure 4f–g represents PhP propagation at ω = 890 cm^–1^. At 890 cm^–1^, FDTD simulations suggest that the PhP propagation along the [100] direction reveals a 24% increase in λ^PhP^ elongation for the 15% index-modulated α-MoO_3_ membrane, compared to the pristine α-MoO_3_ flake. This wavelength elongation, in turn, shifts the dispersion toward the lower momentum region. We run FDTD calculations for other frequencies ranging from 870 cm^–1^ to 905 cm^–1^ and calculate the dispersion for the cases of pristine and thermomechanically processed α-MoO_3_ flakes. In Figure 4h, we show these simulated dispersion curves for the 102 nm α-MoO_3_ pristine flake (dashed black curve) and for a permittivity modulation of 12.5% (dashed orange curve) and 15% (dashed red curve), respectively. The orange and red solid curves represent the dispersion measured by PiFM nanoimaging for hot-pressed samples at 160 °C and 185 °C, respectively. We found agreement between the dispersion curves plotted for the cases of permittivity-modulated FDTD calculations and PiFM-recorded dispersions plotted from OV and strain-induced hot-pressed flakes. This suggests that for a 102 nm pristine α-MoO_3_, a 12.5% and a 15% increase in the static dielectric components along the two *x* and *y* directions can model the response of hot-pressed flakes processed at 185 °C and 160 °C, respectively.

To compare the modulation capability of thermomechanical processing in α-MoO_3_ for phonon-polaritons, we have surveyed common modulation approaches in Supporting Information Figure S4. Our results show that thermomechanical vacancy engineering provides a robust stoichiometric route to permanently reconfiguring PhPs in α-MoO_3_ via coupled oxygen-vacancy formation and strain-driven lattice perturbations, enabling permanent modulation of the dielectric response. Relative to previously reported stoichiometric strategies, this method achieves the largest dispersion shifts while maintaining favorable propagation characteristics, including quality factors and damping rates that remain comparable to or better than other PhP-engineering approaches in α-MoO_3_.

### Outlook

Building on these insights, a broader framework for controlled oxygen-vacancy induction in polar crystals may begin to emerge. Such principles may generalize to other oxygen-rich polar crystals such as α-V_2_O_5_,^53^ β-GaO,^54^ LiV_2_O_5_,^55^ SrTiO_3_,^56^ and their heterostructures.^57^ Our proof-of-concept may help to develop a stoichiometric approach that provides a fundamental route toward controlling the constituents in situ at the interface between two or more general classes of oxygen-rich systems. To explore the scope of the hot-pressing method across various combinations of polaritonic flakes and substrates, we provide a comparative analysis in Supporting Information Figure S16, which may inform future studies. Additionally, in heterostructure platforms, vacancy- and strain-engineered α-MoO_3_ could be integrated with electrically tunable 2D layers to provide complementary control over the local carrier environment. In this context, voltage-driven gating and carrier injection in vdW heterostructures—such as graphene-gated architectures^58–60^ —offer an additional degree of freedom that can operate alongside oxygen-vacancy- and strain-induced modifications of the lattice. Combining intrinsic stoichiometric tuning with external electrostatic control may enable hybrid approaches to modulate phonon-polaritonic behavior across broader spectral ranges and device geometries, expanding the design space for actively reconfigurable polaritonic systems.

## Conclusions

We demonstrate a thermomechanical approach to achieve highly tunable PhPs in α-MoO_3_ via controlled oxygen vacancy formation and compressive strain, which induce permanent changes in the dielectric function without lithography, external fields, or intercalants. Photoinduced force microscopy reveals an average polariton wavevector shift of Δ*k*/*k* ≈ 0.13 within the lower Reststrahlen band for processing temperatures between 160 and 200 °C. Stoichiometric and structural characterization confirms that the observed tuning arises from the modest vacancy formation together with compressive lattice strain, consistent with our DFT and FDTD analyses. Despite the structural changes, the lifetimes of vacancy-engineered phonon-polaritons are measured to be 1.15 ± 0.29 ps with an average loss in lifetime of only 29% compared to the pristine. Our findings establish thermomechanical vacancy engineering as a viable route for reprogramming MIR light-matter interactions in vdW materials for integrated, nonvolatile polaritonic nanophotonics.

## Methods and Experimental Procedures

### Thermomechanical Processing of α-MoO_3_ Flakes

We mechanically exfoliated high-quality α-MoO_3_ from bulk α-MoO_3_ crystals (2D Semiconductor Inc.) directly onto clean, flat 10 × 10 mm^2^ Si substrates. Following exfoliation, we used another Si substrate for capping the α-MoO_3_. The assembly was then placed in a home-built pressure device to apply mild uniaxial pressure onto the flakes while heating. We then placed the pressure device assembly into a commercial benchtop muffle furnace (Thermo Scientific) and heated it for 30 min at various temperatures in ambient conditions.

### PiFM Nanoimaging Measurements

A Molecular Vista Inc. Vista One microscope was connected to a Block Engineering LaserTune quantum cascade laser (QCL) system, with wavenumber resolution of 0.5 cm^–1^ and a tuning range from 782 to 1920 cm^–1^. During the operation, the microscope utilized noncontact high-frequency cantilevers with Pt/Ir-coated tips from Molecular Vista and operated in sideband mode at a QCL intensity of 5%.

### Raman Microscopy

A confocal Raman microscope (Renishaw Inc.), equipped with an objective lens (Nikon Plan Fluor 50×, NA = 0.4) and a 532 nm laser source (22 mW, 50× objective, spot size 1 μm) in ambient conditions, was used to acquire micro-Raman and PL spectra from pristine and hot-pressed α-MoO_3_ flakes on Si substrates.

### Stoichiometric Characterizations

We used Quanta 3D FIB-SEM to capture SEM images of pre- and post-processed α-MoO_3_ flakes. The X-ray photoelectron spectroscopy (XPS) was performed by using AXIS Supra by Kratos analytical instrument with a dual anode A1 Kα (1487.6 eV) monochromatic X-ray source, and high spatial resolution of 0.1 μm to characterize the chemical composition and stoichiometry of hot-pressed α-MoO_3_. The binding energy calibration was performed by using C 1s peak (284.8 eV) as a reference value.

### FDTD Simulations

We performed full-wave electromagnetic simulations using a FDTD method as implemented in Ansys Lumerical FDTD software. The boundary conditions along the *x*-, *y*- and *z*-directions were set with perfectly matched layers. To excite and launch highly confined PhPs, we use a point-like electric dipole source polarized along the *z*-direction. The dipole was positioned at a height of 100–150 nm from the uppermost surface of the target α-MoO_3_. We record the Re­(*E*_*z*_) at a distance of 10 nm on top of the uppermost surface. In this method, we scan the dipole across the topmost surface of the flake and record Re­(*E*_*z*_(*x*, *y*)). We extract the dispersion contours using a fast Fourier transform of the recorded Re­(*E*_*z*_). Here, it is noted that in PiFM, the QCL uses p-polarized light for illuminating the metal tip kept 30–50 nm away from the top surface of the target flakes. As a result, the collected polaritonic electric fields are predominantly applied along the *z*-direction. Such a dipole excitation mechanism allows us to mimic the PiFM excitation and collection scheme. The permittivity of the α-MoO_3_ flakes has been modeled with a Drude–Lorentz model as reported in previous reports.^6^ For the details of the permittivity modeling in the cases of pristine and hot-pressed α-MoO_3_ flakes, see Supporting Information Figure S6–S8.

### DFT Calculations

DFT calculations are performed using the Vienna ab initio Simulation Package (VASP)^61^ with projector augmented wave (PAW) pseudopotentials Mo (4s^2^ 4p^6^ 5s^1^ 4d^5^) and O (2s^2^ 2p^4^),^62^ and employing the vdW-DF approach, which accounts for the dispersion interaction between the structure layers, optimizing the unit cell with various exchange functionals paired with vdW-DF. Density functional theory (DFT) calculations were performed using a 3 × 3 × 1 supercell with a plane-wave energy cutoff of 700 eV and a 4 × 4 × 2 Γ-centered *k*-point mesh for Brillouin zone sampling.^63^ All structures were fully optimized until the residual forces on the ions were less than 0.01 eV for stoichiometric cells and 0.05 eV for defect cells.

## Supplementary Material



## Notes

Details of PiFM characterization and dispersion calculations; details of FDTD simulations; details of structural and chemical stoichiometric characterization; details of Raman and XPS spectral analysis; details of electrical characterization and details of DFT calculations for various pristine and hot-pressed α-MoO_3_ samples (PDF)
