# Supplementary material for: Vacancy-Engineered Phonon Polaritons in a van der Waals Crystal
Source: ACS Nano. 2026 May 15;20(22):15905–15. doi: 10.1021/acsnano.5c20443 (PMC13255253; doi:10.1021/acsnano.5c20443)
Supplement: Supplementary file 1 [file nn5c20443_si_001.pdf]

Vacancy-Engineered Phonon Polaritons in a van der Waals Crystal. Supplementary Information

Mashnoon Alam Sakib,[†,§] Naveed Hussain,[*,†,§] Mariia Stepanova,[†] William Harris,[‡] Joshua J. Bocanegra,[‡] Juan Diego Sanchez,[¶] Camilo Velez Cuervo,[¶] Ruqian Wu,[‡] H. Kumar Wickramasinghe,[†] and Maxim R. Shcherbakov[*,†]

†*Department of Electrical Engineering and Computer Science, University of California, Irvine, CA 92697, USA*

‡*Department of Physics and Astronomy, University of California, Irvine, CA 92697, USA*

¶*Department of Mechanical and Aerospace Engineering, University of California, Irvine, CA 92697, USA*

§*Contributed equally to this work*

E-mail: naveed.hussain@toyota.com; maxim.shcherbakov@uci.edu

Results and discussion

Nanoimaging PhPs in hot-pressed α-MoO_3 using PiFM

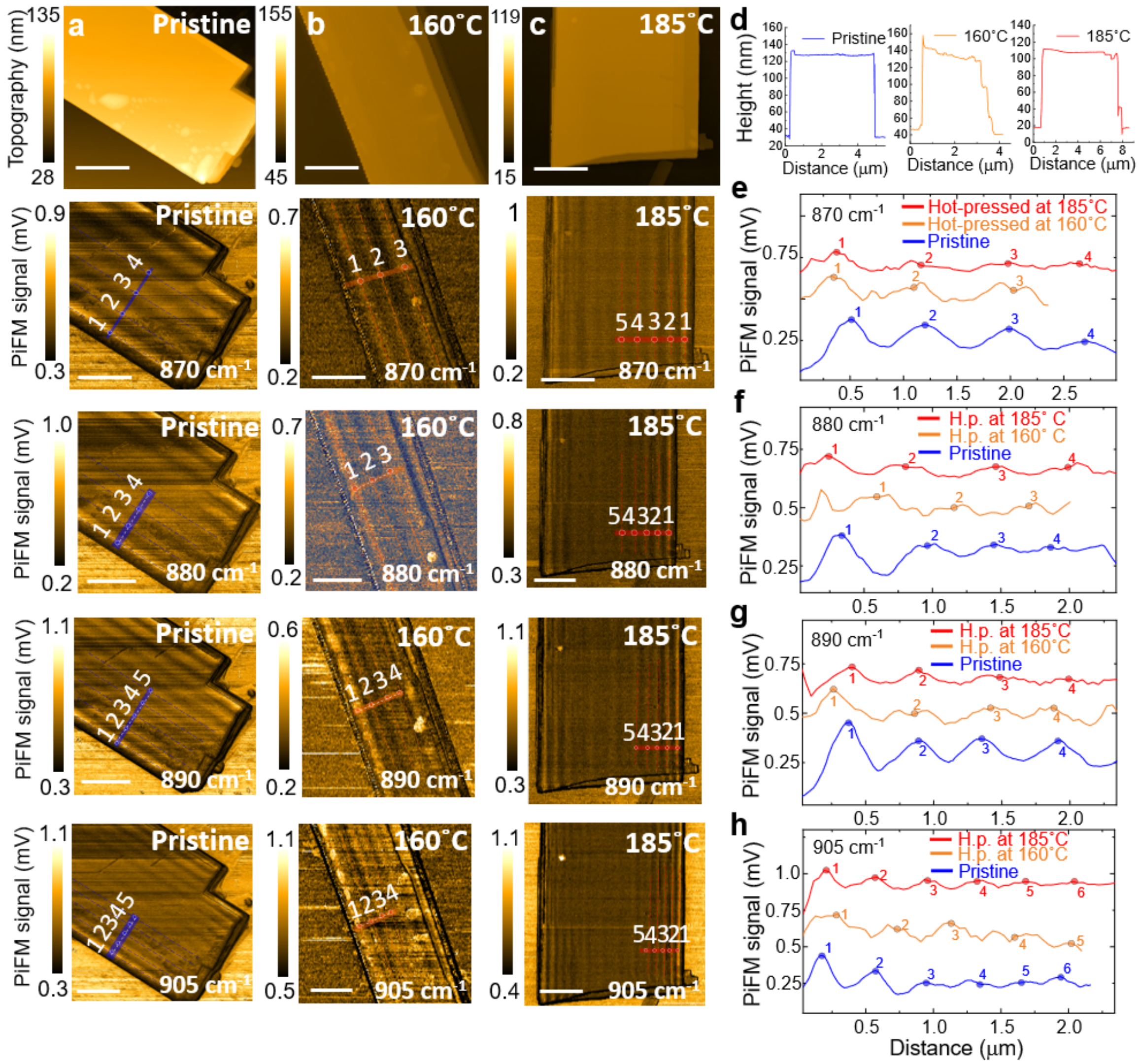

Fig. S1: Nanoimaging PhPs in pristine and hot-pressed α-MoO_3 on silicon substrate. Height profiles shown for a, pristine (t_{pr} = 107 nm), b, 160° C ($t_{h.p.160°C}$ = 102 nm) and c, 185° C ($t_{h.p.185°C}$ = 102 nm) hot-pressed α-MoO_3 flakes. The scales represent 1 μm. Corresponding PiFM images are shown for frequencies 870, 880, 890 and 905 cm^{-1} along the columns under a, for pristine, b, for 160°C and c, for 185°C h.p. α-MoO_3 flakes. d, 2D topography (height) profiles of the flakes e-h, The extraced PhPs lineprofiles are overlapped at similar frequencies for the pristine and h.p. α-MoO_3 flakes at e, 870 cm^{-1}, f, 880 cm^{-1}, g, 890 cm^{-1}, and h, 905 cm^{-1}. e-h, shows that hot-pressing elongates the PhP wavelength (λ_{PhP}) at each of these frequencies shown suggesting the α-MoO_3 L-RB PhP dispersion to be tuned towards lower momenta regime.

Extraction of PhP propagation characteristics in α-MoO_3

We performed the lifetime calculation of α-MoO_3 PhPs according to τ_x=L_X/v_g. Here, the group velocity is calculated from the experimentally recorded dispersion curves (shown in Fig. 2c-f). Group velocity is defined by $v_g = d\omega/dk_x$. In order to calculate the group velocity of the α-MoO_3 PhPs, we use the first-order derivative of the dispersion curves in Fig. 3b (main text) which we get from the experimental PiFM measurements. We take the derivative along the [100] crystal axis directions since our frequency range of interest lies in the L-RB. We numerically fit the experimental dispersion curve data points by a general potential function. This function is taken to be of $y = ax^b$. Followed by the fitting, to get v_g, we calculate a numerical derivative of the resulting curves.

Moreover, for extracting the propagation length (L_X), we fitted the near-field line profiles of the PhPs. These near-field line profiles represent the real part of the z-component of the electric fields along the crystal direction of [100]. We fitted the captured PiFM signal to an exponentially decaying sinusoidal signal along with a dissipation factor. The model equation upon which we performed our fitting is mentioned below:[S1]

$$y = y_0 + Ae^{-x/t_0} \sin\left(\pi \frac{x - x_c}{w}\right), \tag{1}$$

where $A > 0$, $W > 0$ and $t_0 > 0$. For the fitting procedure, we used the Levenberg-Marquardt iteration algorithm. After fitting is performed, we can calculate from the fitted parameter t_0 which represents an estimate of L_X of PhPs.

PiFM PhP characterization of pristine and h.p. α-MoO_3 flakes at 160°C and 185°C

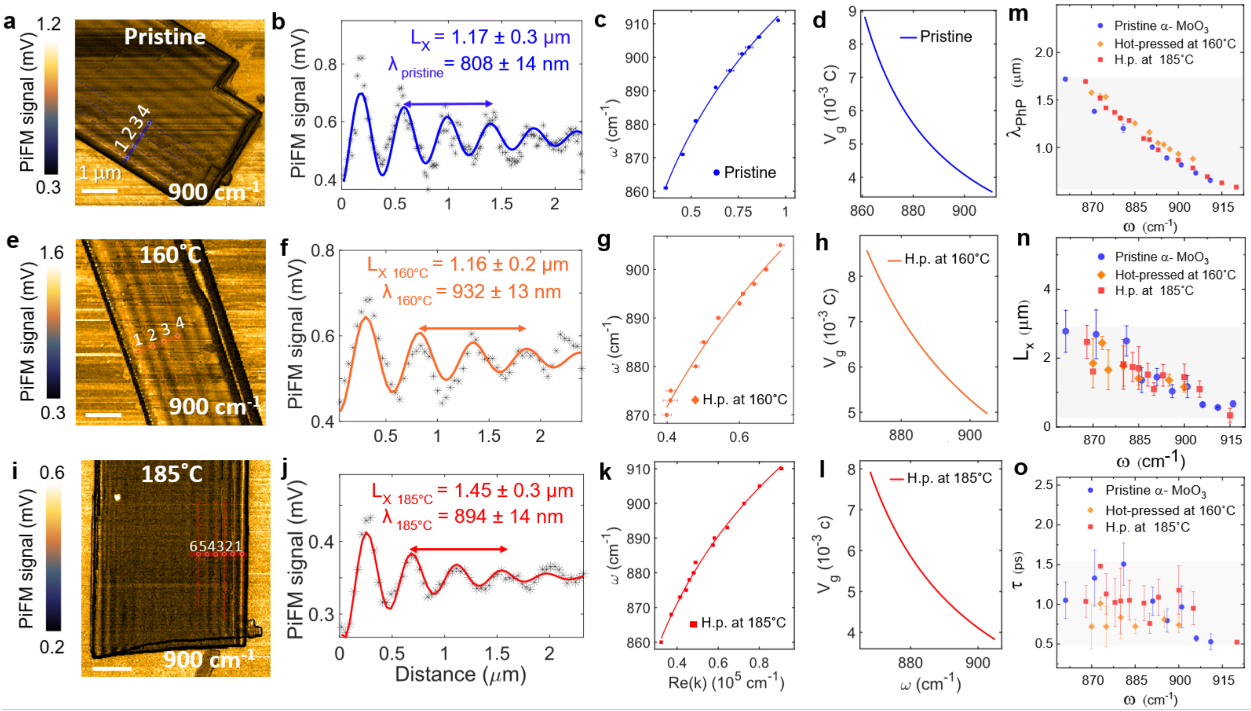

Fig. S2: PhP characterization of pristine and h.p. α-MoO_3 using PiFM. Extracted PhP lineprofiles are fitted to an exponentially-decaying sinusoidal signal model for (a-b) pristine and h.p. α-MoO_3 flakes at (e-f) 160°C and (i-j) 185°C. As a representation, the fittings for 900 cm^{-1} is shown here. The dispersion and group-velocity for each of these cases are shown for (c-d) pristine and h.p. flakes at (g-h) 160°C and (k-l) 185°C, respectively. The key figure of merits used for characterizing the PhP propagation dynamics are PhP wavelength (λ_{PhP}), propagation length (L_X) and lifetime (τ). The comparisons in these FOMs are shown in (m) for λ_{PhP}, (n) for L_X and (o) for τ of pristine and h.p. α-MoO_3 flakes. The error bars of lifetimes were calculated based on the errors of propagation lengths originating from the standard deviation of fitting. Measurements are performed within the lower Reststrahlen band (L-RB) of α-MoO_3, ranging from 865 to 915 cm^{-1}.

PiFM PhP characterization of h.p. α-MoO_3 flake at 200°C

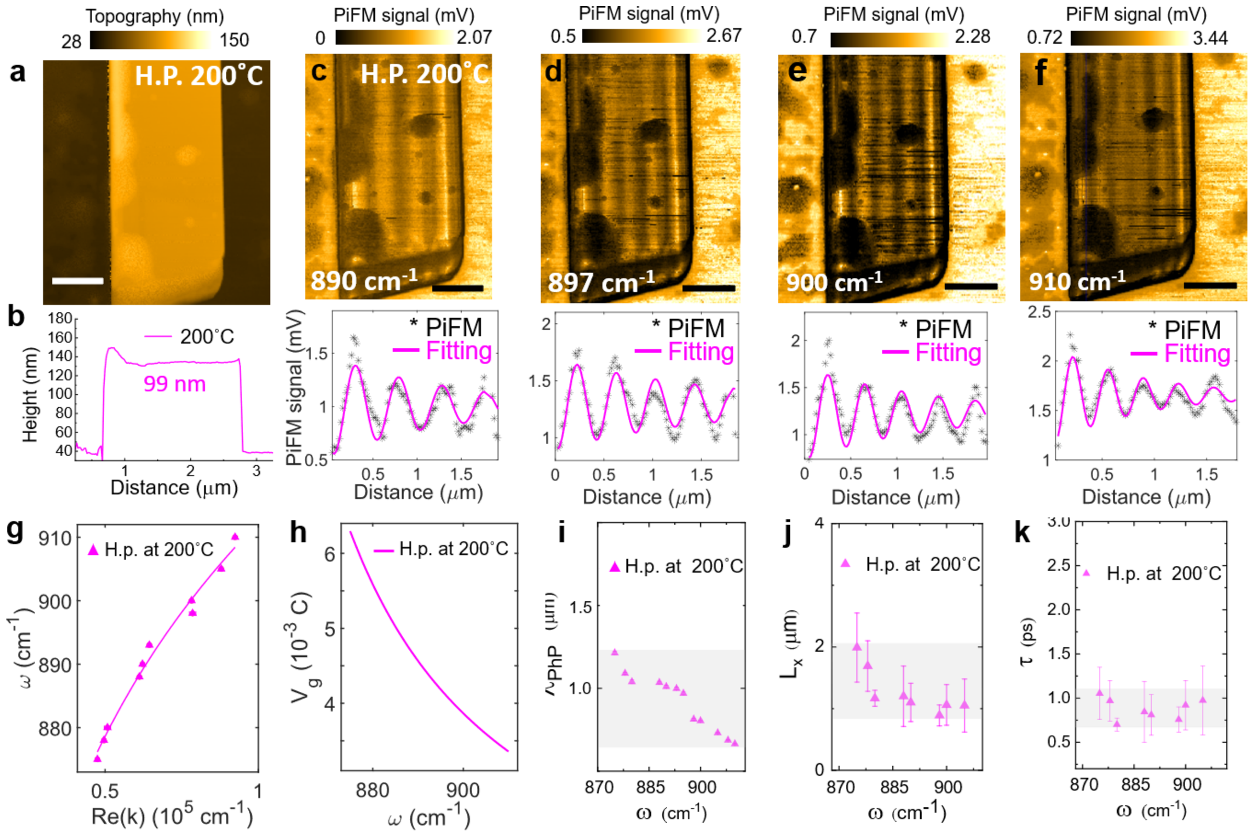

Fig. S3: Nanoimaging PhPs in 200°C hot-pressed α-MoO_3 using PiFM. (a-b) Height profile shown for the measured h.p. flake with thickness, $t_{h.p.200°C} = 99$ nm. Recorded PiFM images are shown for representative frequencies of (c) 890, (d) 897, (e) 900 and (f) 910 cm^{-1}. Corresponding PhP lineprofiles are fitted to an exponentially-decaying sinusoidal function as shown in (c-f). The dispersion and group-velocity for 200° C hot-pressed α-MoO_3 are shown in (g) and (h), respectively. The FOMs for PhP propagation are shown over frequencies in (i-k). The error bars of lifetimes are calculated based on the errors of propagation lengths originating from the standard deviation of fitting. Measurements are performed within the lower Reststrahlen band (L-RB) of α-MoO_3, ranging from 865 to 915 cm^{-1}.

Figure of merits of thermomechanically processed α-MoO_3 flakes

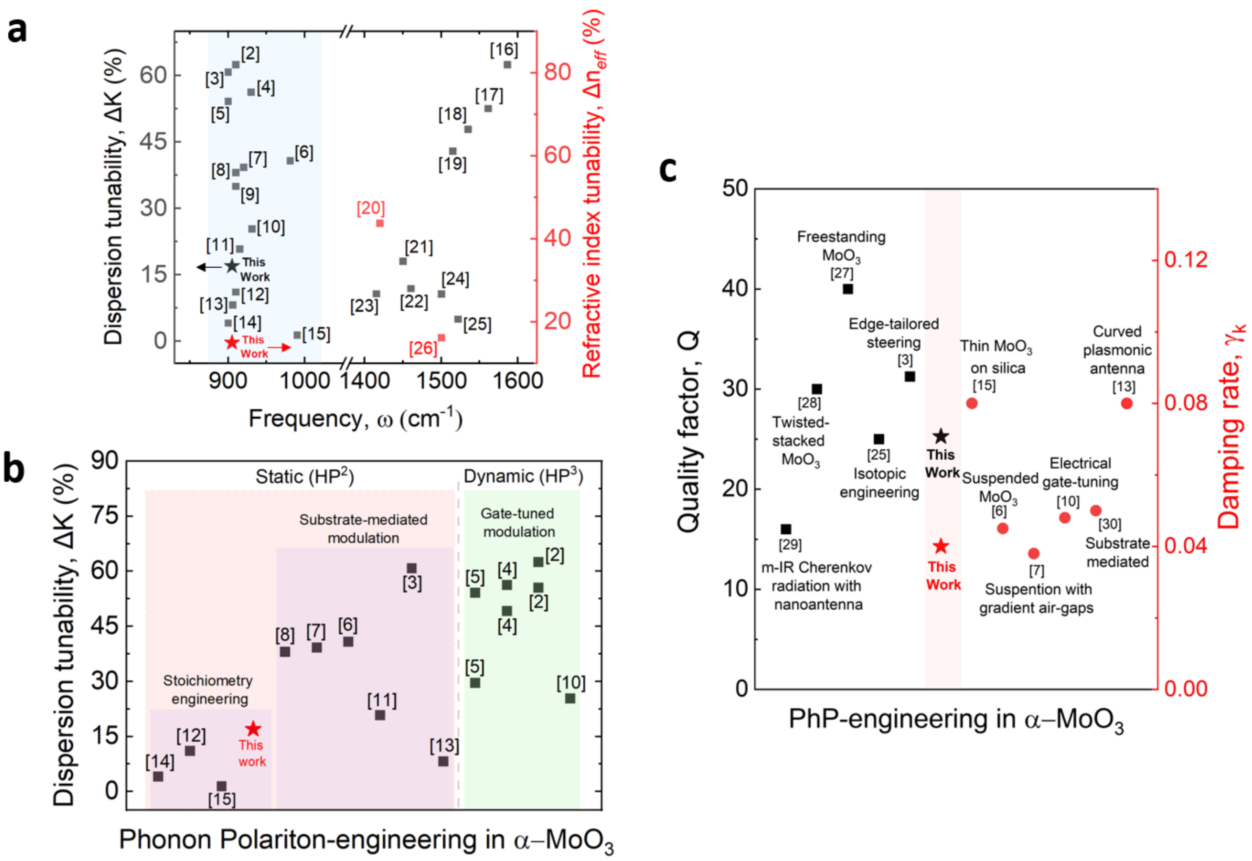

Fig. S4: Figure of merit analysis of thermomechanically-processed α-MoO_3. (a) Dispersion tunability of PhPs extracted from reported works that are related to IR-nanoimaging of PhPs across different van der Waals (vdW) materials. (b-c) Comparison of this work with respect to quality factor (Q) and PhP damping rate (γ_k) with respect to the reported PhP engineering mechanisms[S2–S11,S11–S29] in α-MoO_3.

The figure of merit (FOM) of PhPs in α-MoO_3 are calculated along the [100] crystal direction. For analysis of PhP FOMs, we calculate the the Q-factor (Q) using the supplementary equation 2.[S3,S11,S25]

$$Q = \frac{\mathrm{Re}(\mathrm{k}_x)}{\mathrm{Im}(\mathrm{k}_x)} \tag{2}$$

We calculate the $\mathrm{Re}(\mathrm{k}_x)$ and $\mathrm{Im}(\mathrm{k}_x)$ by fitting the PiFM linescans as mentioned in the pre-

vious section. Here, the Im(k_x) is related to the propagation length (L_X) as follows:[S3,S11,S25]

$$\mathrm{Im}(\mathrm{k}_x) = \frac{1}{2L_X}. \tag{3}$$

Dielectric permittivity modeling of pristine and hot-pressed α-MoO_3 in FDTD

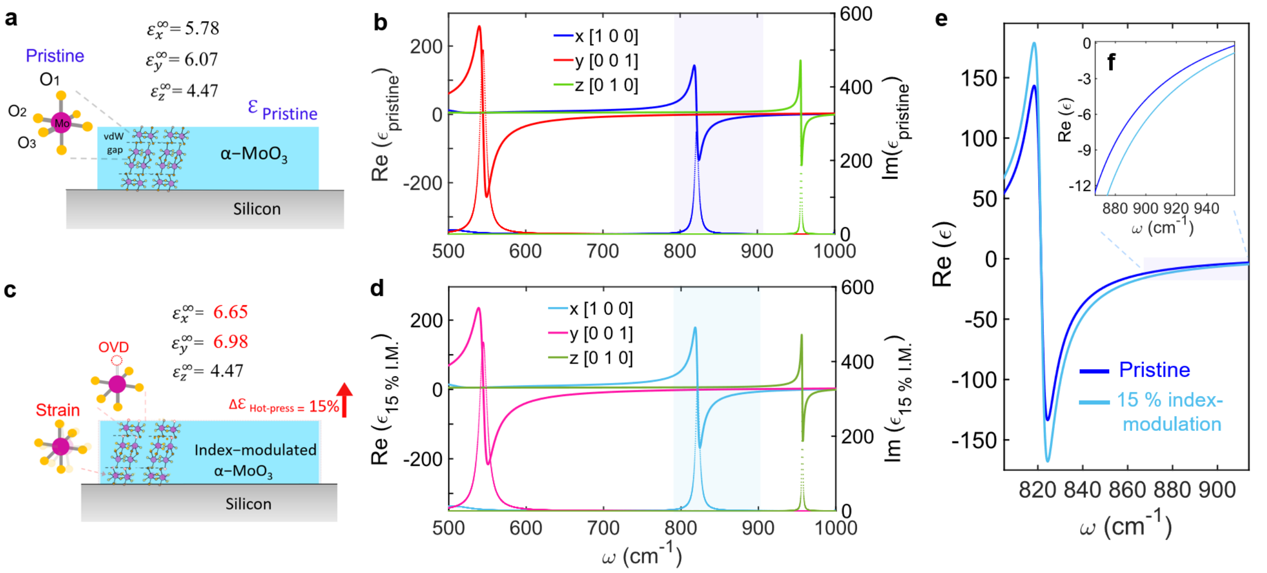

Fig. S5: Dielectric permittivity modeling of pristine and hot-pressed α-MoO_3 in FDTD. (a) Schematic illustration of a pristine α-MoO_3. (b) The dielectric permittivities for this pristine α-MoO_3 model are plotted along the x [100], y [001] and z [010]. The crystallographic axes: [001], [100] and [010], correspond to phonons in each of the Reststrahlen bands (RB_1(red) , RB_2(blue) and RB_3(green), respectively. (c-d) Hot-pressed α-MoO_3 is modeled by introducing the effects of strain and OVD through tuning the static dielectric constants along the x and y crystal-direction. Schematic illustration of index-modulated; h.p. α-MoO_3 model is shown in (c). The dielectric permittivities for this index-modulated α-MoO_3 model are plotted in (d). In (b-d), the region of interest over the L-RB is shown with shaded areas. (e) Changes in the L-RB dispersion relationships (shown in the shaded regions in (b-d)) are overlapped for analyzing the spectral tuning of the L-RB. The inset shows a zoomed-in look into the shaded region of interest from 865-915 cm^{-1} between the pristine and index-modulated α-MoO_3 model.

Since α-MoO_3 is an anisotropic polar dielectric crystal, we model the permittivity by the Lorentz model for the case of coupled oscillators (also known as the TO-LO model).[S1,S21,S30,S31] We use three oscillators for approximating ϵ_x and one oscillator each for the cases of ϵ_y and

ϵ_z, respectively.

$$\epsilon_x(\omega) = \epsilon_x^{\infty} \left(\frac{(\omega_{x1}^{LO})^2 - \omega^2 - i\gamma_{x1}\omega}{(\omega_{x1}^{TO})^2 - \omega^2 - i\gamma_{x1}\omega} \right) \left(\frac{(\omega_{x2}^{LO})^2 - \omega^2 - i\gamma_{x2}\omega}{(\omega_{x2}^{TO})^2 - \omega^2 - i\gamma_{x2}\omega} \right) \left(\frac{(\omega_{x3}^{LO})^2 - \omega^2 - i\gamma_{x3}\omega}{(\omega_{x32}^{TO})^2 - \omega^2 - i\gamma_{x3}\omega} \right) \tag{4}$$

$$\epsilon_y(\omega) = \epsilon_y^{\infty} \left(\frac{(\omega_{y1}^{LO})^2 - \omega^2 - i\gamma_{y1}\omega}{(\omega_{y1}^{TO})^2 - \omega^2 - i\gamma_{y1}\omega} \right) \tag{5}$$

$$\epsilon_z(\omega) = \epsilon_z^{\infty} \left(\frac{(\omega_{z1}^{LO})^2 - \omega^2 - i\gamma_{z1}\omega}{(\omega_{z1}^{TO})^2 - \omega^2 - i\gamma_{z1}\omega} \right) \tag{6}$$

In this Lorentz model, the three principal axes of the α-MoO_3 can be considered in the [100], [001] and [010] directions. Here, the ϵ_x (ω), ϵ_y (ω), and ϵ_z (ω) represent the three principal components of the permittivity tensor, respectively. To generalize this representation, the permittivity tensors can be then denoted by ϵ_i (ω) where the $i = x, y, z$. Here, the static dielectric constant is represented by ϵ_i^{∞}. The LO and TO phonon frequencies along the three directions is represented by ω_{ij}(LO) and ω_{ij}(TO) along the i-th direction with $i = x, y, z$ respectively. Moreover, $\gamma_{ij}(LO)$ represent the damping factor of the Lorentzian line shape derived from the phonon scattering rate. The subscript j here represents the different phonon pairs along the same axis. The parameters used in modeling the dielectric permittivity model of the α-MoO_3 system have been utilized by model fitting the optical response of the material measured from polarized far-field IR spectroscopy.[S30] The static dielectric permittivity tensor components are taken as $\epsilon_x^{\infty} = 5.78$, $\epsilon_y^{\infty} = 6.07$, and $\epsilon_z^{\infty} = 4.47$.

We run density functional theory (DFT) calculations for the hypothesis of thermomechanically index-modulated α-MoO_3. The DFT calculations reflect an increase in the static dielectric constants for a hot-pressed flake. We use FDTD to simulate the influence of lattice strain- and OVD-induced index-modulation over PhPs. The thermomechanical-index modulation is mimicked by tuning the dielectric permittivity. As suggested from our DFT calculations, we increase the static dielectric permittivity tensor components, ϵ_x^{∞} and ϵ_y^{∞}, along the [100] and [001] directions, respectively. This modifies the L-RB for the hot-pressed

α-MoO_3. We show this modified L-RB calculation for the 160°C hot-pressed case. For this case, the static dielectric constants are used as, $\epsilon_x^{h.p.} = 1.15 \times \epsilon_x^{\infty} = 6.65$ and $\epsilon_y^{h.p.} = 1.15 \times \epsilon_y^{\infty} = 6.98$. It is worth noting that the tensor component along the [010] direction shows insignificant changes from the DFT calculations, as the van der Waals gap limits interlayer interaction. As a result, the static dielectric constant ϵ_z^{∞} remains unchanged. Further, we chose to neglect the LO/TO frequency shifts in our modeling, as such calculations for large defect structures are computationally expensive and may not be physically relevant given the periodic nature of the defects.

PiFM and FDTD for a 107 nm pristine α-MoO_3 flakes on a silicon substrate.

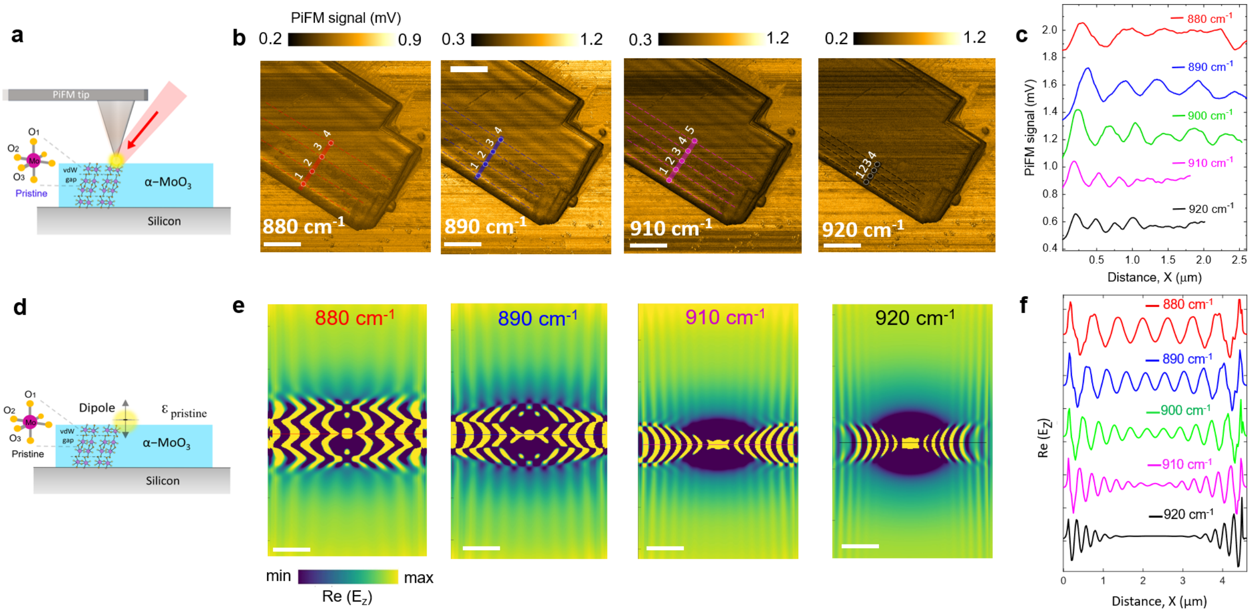

Fig. S6: PiFM and FDTD for a 107 nm pristine α-MoO_3 flakes on a silicon substrate. (a) Schematic illustration of PiFM (b) PiFM images and (c) extracted PhP linescans with for excitations with frequencies from 860 – 920 cm^{-1} within the L-RB of α-MoO_3. The scale bar represents 2 μm. (d) Schematic illustration of dipole-launched PhPs for FDTD (d) Numerically simulated out-of-plane component of electric-field ($Re(E_z)$) for frequencies from 860 – 920 cm^{-1} and (e) corresponding linescan profiles for the PhPs

Analytical model for tunable dispersion of thermomechanically-engineered α-MoO_3

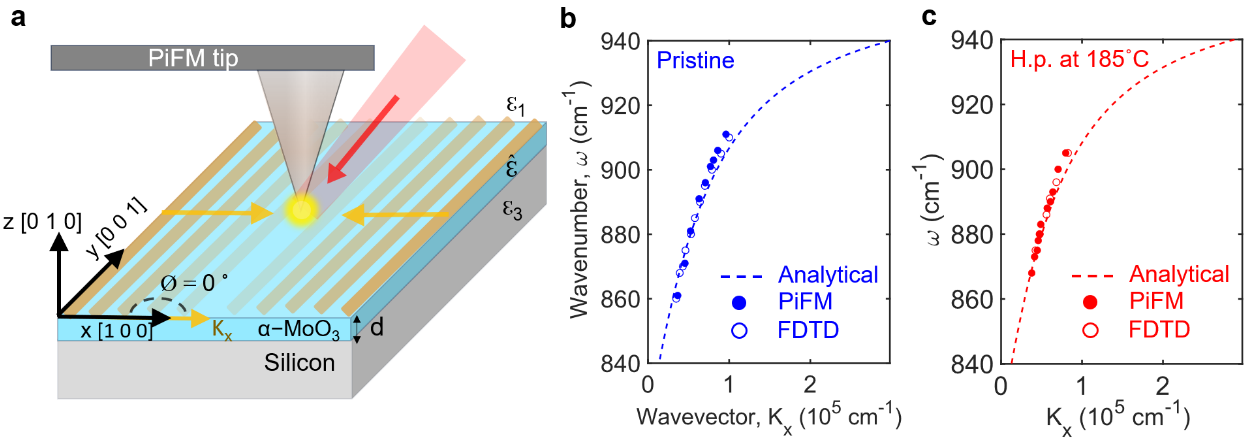

Fig. S7: Analytical modeling for dispersion of PhPs in thermomechanically-engineered α-MoO_3 slabs (a) Schematics of the α-MoO_3 biaxial slab on a silicon substrate. Crystal axes [1 0 0] (directed along x) and [0 0 1] (directed along y) belong to a plane parallel to the faces of the α-MoO_3 slab. The crystal axis [0 1 0] coincides with the z axis. To generalize the hyperbolic L-RB mode propagation, the wave vector K propagates at an angle ϕ with respect to the x (along [1 0 0]) axis. Here, PiFM tip-launched PhP modes originate from the dipole-like excitation source that is originated at the tip due to the mid-IR L-RB excitation frequencies. The PhP modes propagate towards the edge of the flake and gets reflected from the edge. This creates the PhP fringe pattern that propagates the PhP modes along the x [1 0 0] direction. (b-c) Analytically calculated (dashed lines) dispersion relationships are plotted along with PiFM (solid circles) and full-wave FDTD calculations (empty circles) for (b) pristine (blue) and (c) 185° C hot-pressed α-MoO_3 flakes (red), respectively.

we consider a α-MoO_3 slab as a biaxial medium with a general dielectric permittivity tensor $\hat{\epsilon}$. In general the $\hat{\epsilon}$ takes a form of[S31]

$$\hat{\epsilon}(\omega) = \begin{pmatrix} \epsilon_x(\omega) & 0 & 0 \\ 0 & \epsilon_y(\omega) & 0 \\ 0 & 0 & \epsilon_z(\omega) \end{pmatrix}. \tag{7}$$

In Fig. S7 (a), the schematic shows the PhP modes launched by the PiFM tip as it is excited with mid-IR L-RB excitation frequencies. Here, $k_0 = \omega/c$ is the free-space wave vector. Within the L-RB band, k_x makes an angle of $\phi = 0°$ with PhP modes that is

propagating along the direction x, the [100] axis. Here, $k_{x,y}$ is the in-plane momentum along the crystal axes x (specifically along [100]) and y (specifically along [001]) direction, respectively.

For the fundamental modes within the L-RB, the PhP fringes parallel to the [010] direction satisfy the Fabry-Pérot quantization condition.[S1,S6,S31] In general, we calculate the wavevector that satisfies the Fabry-Pérot condition based on an analytical solution as,[S31]

$$q = \frac{\rho}{dK_0}\left[\arctan\frac{\epsilon_1\rho}{\epsilon_z} + \arctan\frac{\epsilon_3\rho}{\epsilon_z} + \pi l\right] \tag{8}$$

Here, $l = 0$ for the case of the fundamental mode propagation. Moreover, $q_{x,y}$ are the in-plane components of the normalized wave vector in the form of $q_{x,y} = k_{x,y}/k_0$. ρ can be defined as

$$\rho = i\sqrt{\frac{\epsilon_z q^2}{\epsilon_x {q_x}^2 + \epsilon_y {q_y}^2}} = i\sqrt{\frac{\epsilon_z}{\epsilon_x \cos^2\phi + \epsilon_y \sin^2\phi}}. \tag{9}$$

For the range of L-RB excitation frequencies with $\phi = 0°$, equation (9) takes a form of

$$\rho = i\sqrt{\frac{\epsilon_z}{\epsilon_x}}. \tag{10}$$

SEM, Raman and topography characterization of pristine and hot-pressed α-MoO_3

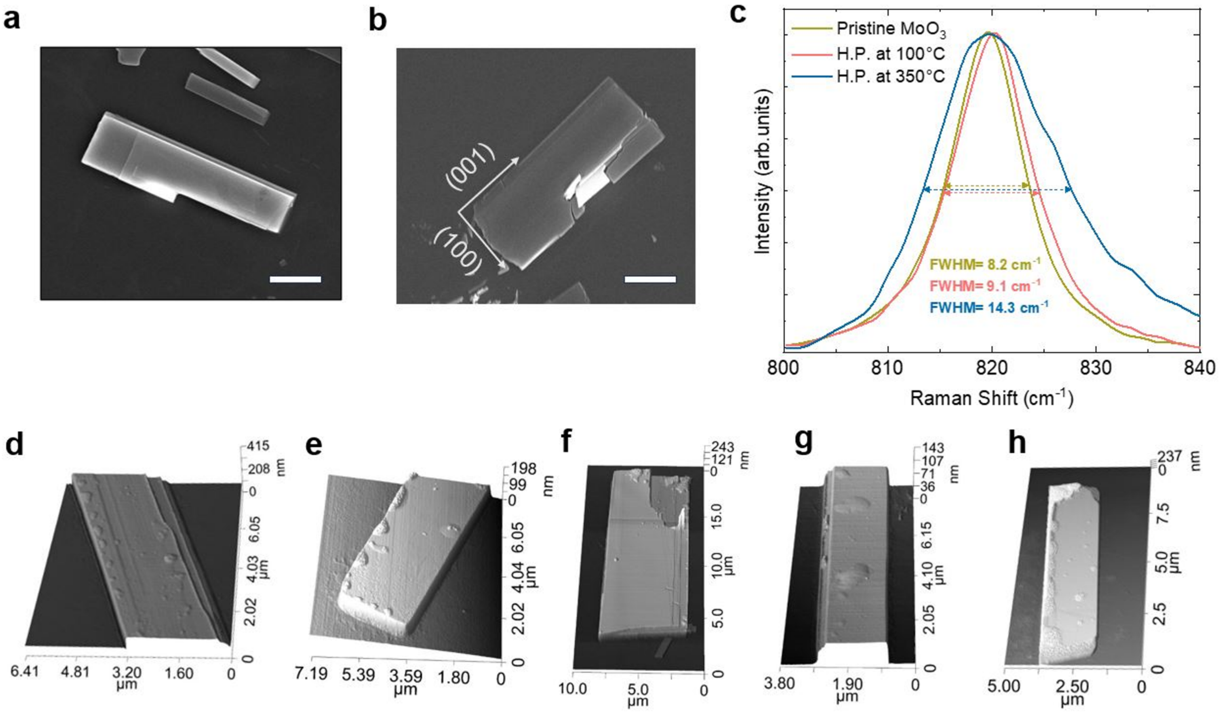

Fig. S8: SEM, Raman characterization and 3D topography profiles of pristine and hot-pressed α-MoO_3. a,b, SEM image of an exfoliated (a) pristine α-MoO_3 flake and (b) hot-pressed flake with its crystal orientation. The scale bar is 10 μm. c, Comparison of the FWHMs of $A_g/B_{1\,g}$ phonon mode of pristine and h.p. α-MoO_3 at 150°C and 350°C. The increase in FWHM with increase in h.p. temperature indicates the introduction of vacancy defect states in α-MoO_3. d-h, 3D topography profiles of hot-pressed α-MoO_3 flakes; extracted from PiFM experiments. These 3D topography profiles suggest no signature of any visible large-scale defects in hot-pressed α-MoO_3 flakes at (d-e) 160°C , (f-g) 185°C and up to (h) 200°C.

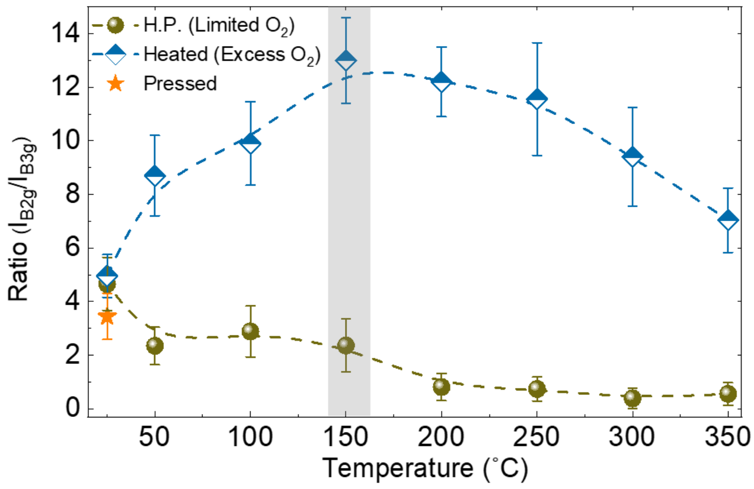

Figure S9: Variation in the ratio of Raman intensities captured at B_{2g} (284 cm^{-1}) and B_{3g} (291 cm^{-1}) mode, $\varsigma = B_{2g}/B_{3g}$ obtained from (green) hot-pressed (with Si substrate encapsulation (limited oxygen in the surrounding environment)) and (blue) heated (without any encapsulation (in air)), shows dissimilar behaviors.

X-ray photoelectron spectroscopy (XPS) survey

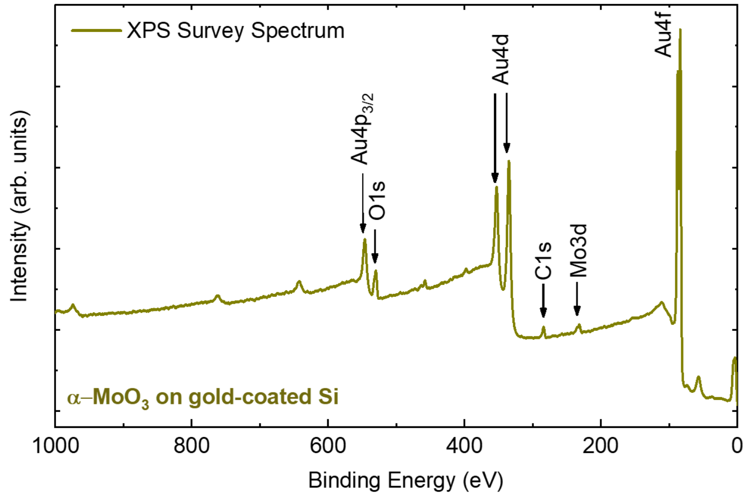

Fig. S10: X-ray photoelectron spectroscopy (XPS) survey spectrum of h.p. α-MoO_3 at 350°C on gold-coated substrate. The gold-coated substrate was used to obtain a conductive surface for better signal acquisition during XPS experiments.

XPS-mapped reduced oxidation state in hot-pressed α-MoO_3

Type	25 ˚C (Fitted Area)			150 ˚C			250 ˚C			350 ˚C		
	Mo^{6+}	Mo^{5+}	Stoichiometric content	Mo^{6+}	Mo^{5+}	Stoichiometric content	Mo^{6+}	Mo^{5+}	Stoichiometric content	Mo^{6+}	Mo^{5+}	Stoichiometric content
$3d_{3/2}$	174 ($3d_{3/2}$)	35.7 ($3d_{3/2}$)	17 % (Mo^{5+}) 82.9 % (Mo^{6+})	2752 ($3d_{3/2}$)	1300 ($3d_{3/2}$)	32.1% (Mo^{5+}) 67.1 (Mo^{6+})	2000 ($3d_{3/2}$)	2150 ($3d_{3/2}$)	51.8(Mo^{5+}) 48.2 (Mo^{6+})	1212 ($3d_{3/2}$)	1814 ($3d_{3/2}$)	59.9 % (Mo^{5+}) 40.05 % (Mo^{6+})
$3d_{5/2}$	275.9 ($3d_{5/2}$)	72.1 ($3d_{5/2}$)	20.4 % (Mo^{5+}) 79.5 % (Mo^{6+})	3535 ($3d_{5/2}$)	800 ($3d_{5/2}$)	18.6% (Mo^{5+}) 81.4 (Mo^{6+})	2150 ($3d_{5/2}$)	1600 ($3d_{5/2}$)	42.6% (Mo^{5+}) 57.4 (Mo^{6+})	1492 ($3d_{5/2}$)	2036 ($3d_{5/2}$)	57.4 % (Mo^{5+}) 42.6 % (Mo^{6+})

Fig. S11: XPS-mapped reduced oxidation state in hot-pressed α-MoO_3 is extracted by spectral peak-fitting of the XPS elemental intensity map.

DFT calculations

DFT calculations were performed using the Vienna Ab initio Simulation Package (VASP)[S32,S33] with projector augmented wave (PAW) pseudopotentials for Mo (4s2 4p6 5s1 4d5) and O (2s2 2p4). The vdW-DF approach was employed to account for dispersion interactions between the layers. A Hubbard U term of 5 eV was applied to Mo to better account for the localized d-electrons. Convergence was achieved with an energy cutoff of 700 eV for the plane-wave basis set with a 9×9×3 gamma-centered k-mesh[S34–S36] for stoichiometric cell calculations and 4x4x3 for the 3x3x1 super-cell structures. All structures were optimized until the residual forces on the ions were less than 0.01 eV for stoichiometric cells and 0.05 eV for defect cells. The static dielectrics are obtained using density functional perturbation theory within VASP.[S35,S36]

DFT calculations of OVD-induced index modulation in α-MoO_3

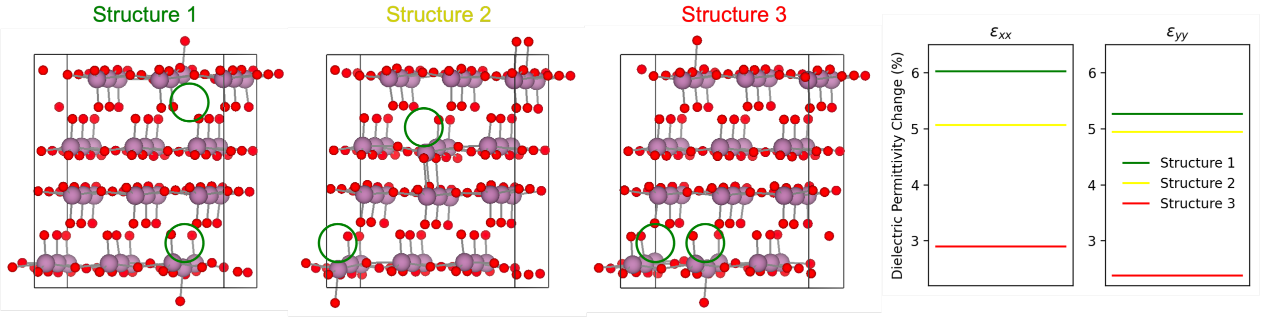

Fig. S12: Structural and dielectric changes in α-MoO_3 with two O_1 site defects placed at different relative positions near the vdW gap. Structures 1, 2, and 3 correspond to distinct randomly selected defect configurations. The dielectric permittivity change (in %) relative to the pristine structure is shown for the ϵ_{xx} and ϵ_{yy} components, highlighting the impact of defect placement on the material's dielectric response. In particular, Structure 3, where vacancies are nearby and located on the same layer, exhibits much smaller modulation, while Structures 1 and 2 yield similar results. Structure 1 is used in the main text as it is less effected by vacancy proximity.

DFT band structure of strained α-MoO_3

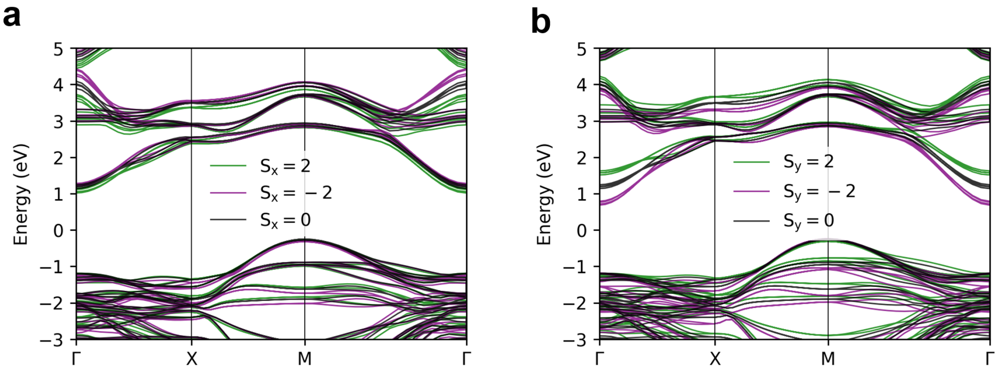

Fig. S13: Band structure of strained α-MoO_3 for positive and negative strains. The left panel shows results for strain along x ($S_x = 2$, $S_x = -2$, and $S_x = 0$), while the right panel shows strain along y ($S_y = 2$, $S_y = -2$, and $S_y = 0$). Notably, the bands near the Γ-point exhibit significant shifts under S_y strain, altering the band gap. This shift correlates with changes in the static dielectric constant, as noted in the main text, where a smaller band gap leads to larger static dielectric.

Current–voltage (I–V) characteristics for pristine and hot-pressed α-MoO_3 flakes

The I-V characteristics are recorded at room temperature under ambient lighting conditions. A Signatone 1160 series probe station built with tungsten tips to establish contact with the devices. The station gets connected to a Keithley 4200A-SCS parameter analyzer mainframe. The I-V measurement results are shown in Fig. S14. Standard micro-fabrication processes involved sequential processes such as UV photolithography, pattern development, electron beam evaporation, and lift-off techniques to pattern interdigitated gold electrodes followed by the PDMS-assisted transfer of pristine and hot-pressed α-$MoO_{3\text{-x}}$ samples onto the gold electrodes.[S37,S38]

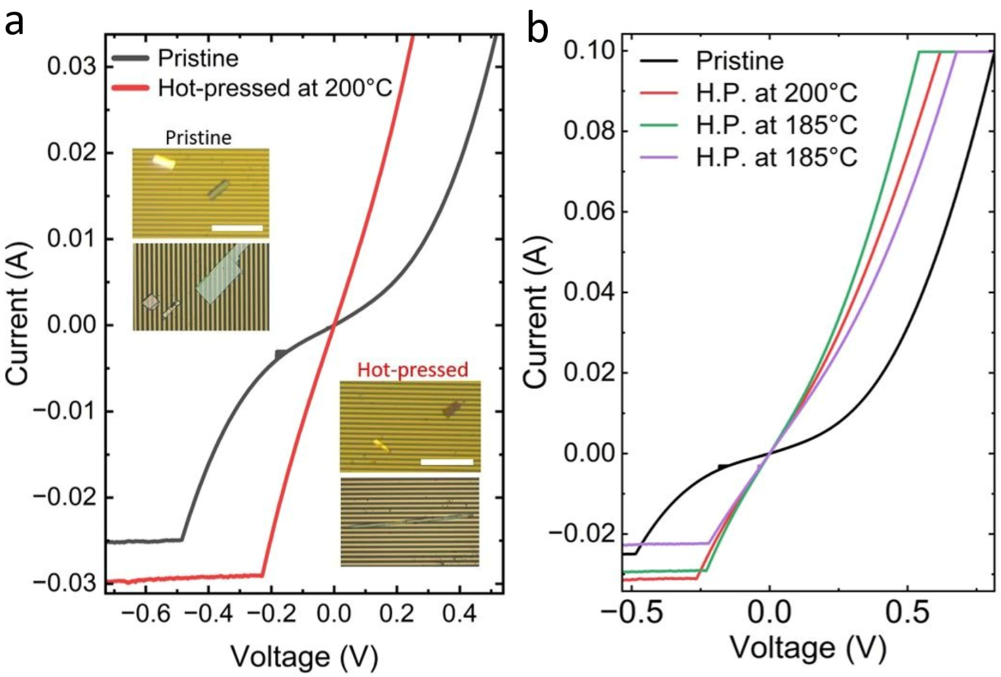

Fig. S14: Current-voltage characteristics for (a) pristine α-MoO_3 and (b) OV-induced hot-pressed α-$MoO_{3\text{-x}}$ based devices.

For both pristine and hot-pressed samples, the I–V characteristics exhibit Schottky-diode-like nonlinear behavior.[S39–S41] Upon fitting the forward bias region using a standard Schottky diode model,[S41] we extracted the ideality factor (n), saturation current (I_s), and series resistance (R_s), which help us to correlate changes in charge carrier concentrations due to

hot-pressing. Fig. S15 shows that, in the forward-bias regime (small-signal series resistance), the hot-pressed $MoO_{3\text{-x}}$ samples exhibit a 50% reduction in resistance of 12.5 ± 0.3 Ω compared to 24.9 ± 2.7 Ω for pristine α-MoO_3. This 50% resistance decrease can only occur if there is an increase in the number of charge carrier concentrations by a factor of ≈ 2.[S39–S41] Additionally, the I-V behavior suggests that n and I_s increase for the hot-pressed $MoO_{3\text{-x}}$ samples compared to the pristine samples. The simultaneous increase in n and I_s, together with the reduction in resistance, suggests that diffusion-dominated transport mechanism (in pristine α-MoO_3) gets modified to a recombination-dominated behavior after hot-pressing (in sub-stoichiometric $MoO_{3\text{-x}}$). This suggests consistency with defect-assisted leakage via oxygen vacancies. Importantly, both pristine and hot-pressed samples (two at 185 °C and one at 200 °C) retain a nonlinear Schottky-like I–V response, with no linear (Ohmic) regime observed.[S41] This also suggests that hot-pressing does not induce a phase transition to metallic MoO_2, and the flakes remain in the sub-stoichiometric $MoO_{3\text{-x}}$ regime.

In addition, throughout Raman and XPS measurements, we did not observe any phase transformation of α-MoO_3 to MoO_2 through the hot-pressing method. We used Raman, XPS, PiFM experiments and utilized DFT and FDTD calculations to show that up to 200 °C, hot-pressing is estimated to introduce 1-2% oxygen vacancies that produce sub-stoichiometric α-$MoO_{3\text{-x}}$. For our experiments, up to 200 °C, with maximum 2% OVs, the pristine α-MoO_3 is expected to become a sub-stoichiometric α-$MoO_{2.94}$. This estimation has also been found to agree well with recently reported work exploring oxygen-poor environments to induce OVs and found 1.4% oxygen vacancies induced within α-$MoO_{3\text{-x}}$.[S42] Moreover, previously reported experiments recorded pristine α-MoO_3 flakes to have charge carrier concentrations values around 6.8×10^{17} cm^{-3}.[S43] Here, we are considering the highest free carrier concentration that has been experimentally recorded in pristine α-MoO_3; for example, in other sources, the charge carrier concentration in pristine α-MoO_3 has also been recorded at $\approx 10^{14}$ cm^{-3}, which is 2-3 orders of magnitude lower when compared to the pristine charge carrier density that we consider.[S44] Furthermore, it was previously observed that the charge carrier density

can increase to 3.7×10^{18} cm^{-3} for x as high as 0.2.[S43]

Taken together, these results suggest that the maximum (worst-case) estimated free carrier concentration in our cases could range from 6.8×10^{17} cm^{-3} to up to 1.4×10^{18} cm^{-3}. These values will be used in the next section to estimate the largest free-carrier-induced correction to the dielectric permittivity to rule out plasma-induced PhP tunability.

Drude-modified dielectric permittivity with oxygen-vacancy defects

The frequency dependent infrared dielectric permittivity for a polar dielectric material modified by the presence of free carriers is given by:[S45–S49]

$$\epsilon\left(\omega, N_j\right) = \epsilon_\infty \left(1 + \frac{\omega_{LO}^2 - \omega_{TO}^2}{\omega_{TO}^2 - \omega^2 - i\gamma\omega}\right) - \left(\frac{\omega_P^2}{\omega^2 + i\omega\Gamma}\right). \tag{11}$$

Here, the first and second terms account for the optical phonon contribution and the Drude-type free carrier contribution that arises from significant charge carrier concentration. For hot-pressed α-$MoO_{3\text{-x}}$, oxygen vacancies donate additional electrons to the lattice and support the recombination-dominated Schottky-diode-like nonlinear I-V behavior. Therefore, we can assume that $N_j \approx N_e$, and the hole contribution can be neglected.[S43,S45,S48,S50–S53] In the Drude term, $\omega_p(j)$ is the plasma frequency, Γ_j is the collision rate of the free carriers, both depending on the free-carrier density N_j via:[S45–S49]

$$\omega_P^{(j)} = \sqrt{\frac{N_j e^2}{\epsilon_0 m^*}}, \tag{12}$$

$$\Gamma_j = \frac{e}{m^* \mu_j(N_j)}. \tag{13}$$

Here, electron charge e, effective mass of the free carrier m^*, carrier mobility μ_j and vacuum permittivity ϵ_0. Γ_j depends indirectly on N_j through the majority charge-carrier

mobility μ_j via Caughey-Thomas expression:[S54]

$$\mu_j = \mu_{min} + \frac{\mu_{max} - \mu_{min}}{1 + \left(\frac{N_j}{N_0}\right)^{\alpha}}. \tag{14}$$

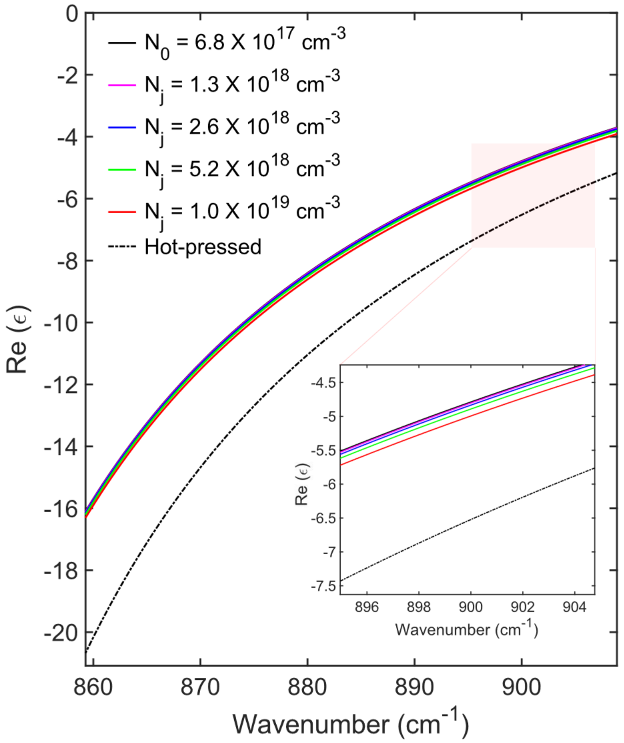

Fig. S15: Calculation of the real component of the dielectric permittivity for OV-induced hot-pressed α-$MoO_{3\text{-x}}$. Inset: the increase in charge carrier concentrations results in negative contributions to the permittivity. The additional 1.5 orders of increase in free electron concentration perturbs the real part of the permittivity along [1 0 0] by a small amount compared to the OV driven changes in ϵ_∞.

The increase in the charge carrier concentration could shift the plasmonic modes to the infrared, including potentially to the mid-infrared. To understand this OV-defect-mediated free-electron contribution, we calculate change in the real part of $\epsilon(\omega)$ for various levels of charge carrier concentrations ranging from 6.8×10^{17} cm^{-3} (for pristine α-MoO_3) to up to 10^{19} cm^{-3} (assuming severely doped $MoO_{3\text{-x}}$). We show these results in Fig. S15. Fig. S15 and its' inset show that an additional 1.5 orders of increase in free electron concentration perturbs the real part of the permittivity along [1 0 0] by an incremental amount at any

given frequency. Fig. S15 also suggests that for the case with charge carriers close to orders of 10^{17} cm^{-3}, the free carriers may not be sufficient to hybridize plasmonic modes with the RB_x of α-MoO_3, where we are nanoimaging the PhPs. The presence of this large IR spectral gap, suggests that — even with modified charge carrier defect sites — the RB-band along [100] would not be able to establish electron-phonon-photon-like hybridization and coupling.

Having mentioned these hypotheses supported by experimentally-calibrated I-V measurements and Drude-modified dielectric permittivity modeling, we conclude that: (1) the I-V behavior does not see qualitative modification to the linear behavior that could have been attributed to formation of conducting phases such as MoO_2, and (2) the phonon-polaritonic modes in sub-stoichiometric α-$MoO_{3\text{-x}}$ are spectrally isolated from free carrier plasma corrections.

Comparative analysis of the CTE-mismatch-driven hot-pressing mechanism across various polaritonic materials and substrates

To explore the scope of the hot-pressing method across various selections of polaritonic flakes and substrates, we provide two tables with the CTE values for the polaritonic materials (i.e., α-MoO_3,[S55] h-BN[S56] and α-V_2O_5[S57]) and for the substrates (i.e., Silicon,[S58] Silica[S59] and quartz[S60]).

Case 01: Silicon - α-MoO_3 - Silicon: There is a two-order-of-magnitude difference in the CTE between Si and α-MoO_3.[S55,S58] This is the largest CTE mismatch among the 5 cases.[S55–S60] This large CTE mismatch ensures mutual thermal expansion and contraction processes along the interfacial planar boundary, offering maximum strain transfer.[S61–S63] This helped create an energetically favorable environment that induced a permanent modification of the stoichiometry of α-MoO_3 with a non-volatile compressive strain profile. Moreover, studies also show that OVs modify the strain and thermal expansion anisotropy, resulting in the modification of CTE.[S64]

Case 02 and 03: Silica (or/ Quartz)- α-V_2O_5 - Silica (or/ Quartz): The relative

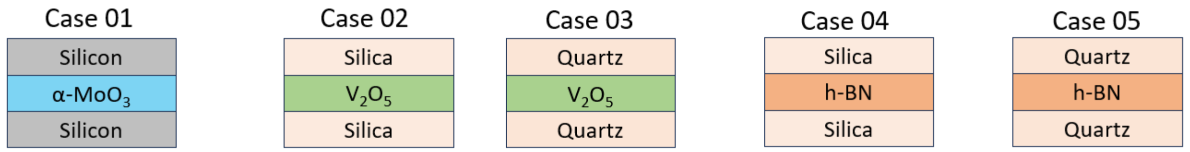

Polaritonic material	CTE (/K)	Ref.
α-MoO_3	$\alpha^{\text{in-plane (x)}}_a$ = - 0.3 x 10^{-4}<br>$\alpha^{\text{out-of-plane(z)}}_b$ = 1.3 x 10^{-4}<br>$\alpha^{\text{in-plane (y)}}_c$ = 5.5 x 10^{-4}	[S55]
α-V_2O_5	α_a = 9.5 x 10^{-6}<br>α_b = 6.9 x 10^{-6}<br>α_c = 3.5 x 10^{-5}	[S57]
h-BN	$\alpha_{\text{in-plane}}$ = - 2.9 x 10^{-6}<br>$\alpha_{\text{out-of-plane}}$ = 4 x 10^{-5}	[S56]

Substrate	CTE (/K)	Ref.
Silicon	α_{si} = 2.6 x 10^{-6}	[S58]
Silica	α_{silica} = 0.55 x 10^{-6}	[S59]
Quartz	$\alpha_{\text{quartz (fused)}}$ = 0.5 x 10^{-6}<br>$\alpha_{\text{quartz (crystal)}}$ = 7.7 x 10^{-6}	[S60]

Fig. S16: Comparative analysis of the CTE-mismatch driven hot-pressing mechanism across polaritonic materials (i.e.,α-MoO_3, h-BN, α-V_2O_5) and substrates (i.e., silicon, silica, quartz).

CTE mismatch in both cases are comparatively lower than that of case 01. Here, the CTEs differ by an order of magnitude along the c-axis of α-V_2O_5.[S57] Interestingly, as α-V_2O_5 is also an oxygen-rich platform,[S57] the relative CTE mismatch for α-V_2O_5 with quartz (or silica) can be a good starting point for exploring thermomechanical effects.[S57,S59,S60] However, we would like to highlight that for silica - α-V_2O_5 (case 02) and for quartz - α-V_2O_5 (case 03), the CTE-mismatch is, respectively, 16 and 20 times smaller in magnitude compared to the case for silicon - α-MoO_3 (case 01).

Case 04 and 05: Silica (or/ Quartz)- h-BN- Silica (or/ Quartz): The relative CTE mismatch in both cases 04 and 05 is the lowest compared to both our experimentally measured cases (case 01) and also compared to cases with α-V_2O_5 (case 02 and 03). Due to the anisotropic thermal conductivity, the in-plane and out-of-plane CTE in h-BN varies.[S56] However, h-BN is also a good thermal conductor and possesses a high thermal shock absorbing capability.[S56,S65] Thus, it is expected that the low processing temperatures that we use (under 200 °C) may not be enough to modulate and enable a vacancy-creation mechanism in h-BN. In addition, unlike α-MoO_3, h-BN does not intrinsically contain oxygen atoms within

its crystal lattice. Therefore, the hot-pressing technique that we have designed to modulate oxygen-vacancy concentrations by restricting or cutting off the oxygen supply may not induce comparable vacancy defects and strain for dispersion modulation in hBN.

Supplementary References

S1. Ma, W.; Alonso-González, P.; Li, S.; Nikitin, A.Y.; Yuan, J.; Martín-Sánchez, J.; Taboada-Gutiérrez, J.; Amenabar, I.; Li, P.; Vélez, S.; Tollan, C; Dai, Z; Zhang, Y.;Sriram, S.; Kalantar-Zadeh, K.; Lee, S.-T.; Hillenbrand, R.; Bao, Q.; In-plane anisotropic and ultra-low-loss polaritons in a natural van der Waals crystal. *Nature* **2018**, *562 (7728)*, 557–562

S2. Hu, H.; Chen, N.; Teng, H.; Yu, R.; Qu, Y.; Sun, J.; Xue, M.; Hu, D.; Wu, B.; Li, C.; Chen, J.; Liu, M.; Sun, Z.; Liu, Y.; Li, P.; Fan, S.; García de Abajo, F.J.; Dai, Q. Doping-driven topological polaritons in graphene/α-MoO_3 heterostructures. *Nature Nanotechnology* **2022**, *17 (9)*, 940–946

S3. Dai, Z.; Hu, G.; Si, G.; Ou, Q.; Zhang, Q.; Balendhran, S.; Rahman, F.; Zhang, B.Y.; Ou, J.Z.; Li, G.; Alú, A.; Qiu, C.W. Edge-oriented and steerable hyperbolic polaritons in anisotropic van der Waals nanocavities. *Nature Communications* **2020**, *11 (1)*, 6086

S4. Álvarez-Pérez, G.; González-Morán, A.; Capote-Robayna, N.; Voronin, K.V.; Duan, J.; Volkov, V.S.; Alonso-González, P.; Nikitin, A.Y.; Active tuning of highly anisotropic phonon polaritons in van der Waals crystal slabs by gated graphene. *ACS Photonics* **2022**, *9 (2)*, 383–390

S5. Zeng, Y.; Ou, Q.; Liu, L.; Zheng, C.; Wang, Z.; Gong, Y.; Liang, X.; Zhang, Y.; Hu, G.; Yang, Z.; Qiu, C.W.; Bao, Q,; Chen, H.; Dai, Z. Tailoring topological transitions of anisotropic polaritons by interface engineering in biaxial crystals. *Nano Letters* **2022**, *22 (10)*, 4260–4268

S6. Shen, J.; Zheng, Z.; Dinh, T.; Wang, C.; Chen, M.; Chen, P.; Ma, Q.; Jarillo-Herrero, P.; Kang, L.; Dai, S.; Hyperbolic phonon polaritons with positive and negative phase velocities in suspended α-MoO_3. *Applied Physics Letters* **2022**, *120 (11)*, 11

S7. Zheng, Z.; Sun, F.; Xu, N.; Huang, W.; Chen, X.; Ke, Y.; Zhan, R.; Chen, H.; and Deng, S.; Tunable hyperbolic phonon polaritons in a suspended Van der Waals α-MoO_3 with gradient gaps. *Advanced Optical Materials* **2022**, *10 (5)*, 2102057

S8. Schwartz, J.J.; Le, S.T.; Krylyuk, S.; Richter, C.A.; Davydov, A.V.; and Centrone, A.; Substrate-mediated hyperbolic phonon polaritons in MoO_3. *Nanophotonics* **2021**, *10 (5)*, 1517–1527

S9. Ruta, F.L.; Kim, B.S.Y.; Sun, Z.; Rizzo, D.J.; McLeod, A.S.; Rajendran, A.; Liu, S.; Millis, A.J.; Hone, J.C.; Basov, D.N.; Surface plasmons induce topological transition in graphene/α-MoO_3 heterostructures. *Nature Communications* **2022**, *13 (1)*, 3719

S10. Zhou, Z.; Song, R.; Xu, J.; Ni, X.; Dang, Z.; Zhao, Z.; Quan, J.; Dong, S.; Hu, W.; Huang, D.; Chen, K.; Wang, Z.; Cheng, X.; Raschke, M.B.; Alú, A.; Jiang, T. Gate-Tuning Hybrid Polaritons in Twisted α-MoO_3/Graphene Heterostructures. *Nano Letters* **2023**, *23 (23)*, 11252–11259

S11. Yang, J.; Tang, J.; Ghasemian, M.B.; Mayyas, M.; Yu, Q.V.; Li, L.H.; Kalantar-Zadeh, K.; High-Q phonon-polaritons in spatially confined freestanding α-MoO_3. *ACS Photonics* **2022**, *9 (3)*, 905–913

S12. Zhao, Y.; Chen, J.; Xue, M.; Chen, R.; Jia, S.; Chen, J.; Bao, L.; Gao, H.; and Chen, J.; Ultralow-loss phonon polaritons in the isotope-enriched α-MoO_3. *Nano Letters* **2022**, *22 (24)*,10208–10215

S13. Zheng, Z.; Jiang, J.; Xu, N.; Wang, X.; Huang, W.; Ke, Y.; Zhang, S.; Chen, H.; and Deng, S.; Controlling and Focusing In-Plane Hyperbolic Phonon Polaritons in α-MoO_3 with a Curved Plasmonic Antenna. *Advanced Materials* **2022**, *34 (6)*,2104164

S14. Wu, Y.; Ou, Q.; Yin, Y.; Li, Y.; Ma, W.; Yu, W.; Liu, G.; and Cui, X.; Bao, X.; Duan, J.; and others; Chemical switching of low-loss phonon polaritons in α-MoO_3 by hydrogen intercalation. *Nature Communications* **2020**, *11 (1)*, 2646

S15. Zheng, Z.; Chen, J.; Wang, Y.; Wang, X.; Chen, X.; Liu, P.; Xu, J.; Xie, W.; Chen, H.; Deng, S.; Xu; N. Highly confined and tunable hyperbolic phonon polaritons in van der Waals semiconducting transition metal oxides. *Advanced Materials* **2018**, *30 (13)*, 1705318

S16. Fali, A.; White, S.T.; Folland, T.G.; He, M.; Aghamiri, N.A.; Liu, S.; Edgar, J.H.; Caldwell, J.D.; Haglund, R.F.; and Abate, Y.; Refractive index-based control of hyperbolic phonon-polariton propagation. *Nano Letters* **2019**, *19 (11)*, 7725–7734

S17. Kim, K.S.; Trajanoski, D.; Ho, K.; Gilburd, L.; Maiti, A.; van der Velden, L.; de Beer, S.; and Walker, G.C.; The effect of adjacent materials on the propagation of phonon polaritons in hexagonal boron nitride. *The Journal of Physical Chemistry Letters* **2017**, *8 (13)*, 2902–2908

S18. Dai, S.; Ma, Q.; Liu, M.K.; Andersen, T.; Fei, Z.; Goldflam, M.D.; Wagner, M.; Watanabe, K.; Taniguchi, T.; Thiemens, M.; Keilmann, F.; Janssen, G.C.A.M; Zhu, S.-E.; Jarillo-Herrero, P.; Fogler, M.M.; Basov, D.N. Graphene on hexagonal boron nitride as a tunable hyperbolic metamaterial. *Nature Nanotechnology* **2015**, *10 (8)*, 682–686

S19. He, M.; Halimi, S.I.; Folland, T.G.; Sunku, S.S.; Liu, S.; Edgar, J.H.; Basov, D.N.; Weiss, S.M.; and Caldwell, J.D.; Guided Mid-IR and Near-IR Light within a Hybrid Hyperbolic-Material/Silicon Waveguide Heterostructure. *Advanced Materials* **2021**, *33 (11)*, 2004305

S20. Chaudhary, K.; Tamagnone, M.; Rezaee, M.; Bediako, D.K.; Ambrosio, A.; Kim, P.; and Capasso, F.; Engineering phonon polaritons in van der Waals heterostructures to enhance in-plane optical anisotropy. *Science Advances* **2019**, *5 (4)*, eaau7171

S21. Folland, T.G.; Fali, A.; White, S.T.; Matson, J.R.; Liu, S.; Aghamiri, N.A.; Edgar, J.H.; Haglund Jr, R.F.; Abate, Y.; and Caldwell, J.D.; Reconfigurable infrared hyperbolic metasurfaces using phase change materials. *Nature Communications* **2018**, *9 (1)*, 4371

S22. Dai, S.; Quan, J.; Hu, G.; Qiu, C.W.; Tao, T.H.; Li, X.; and Alù, A.; Hyperbolic phonon polaritons in suspended hexagonal boron nitride. *Nano Letters* **2018**, *19 (2)*, 1009–1014

S23. Wang, H.; Janzen, E.; Wang, L.; Edgar, J.H.; and Xu, X.G.; Probing mid-infrared phonon polaritons in the aqueous phase. *Nano Letters* **2020**, *20 (5)*, 3986–3991

S24. Virmani, D.; Bylinkin, A.; Dolado, I.; Janzen, E.; Edgar, J.H.; and Hillenbrand, R.; Amplitude-and phase-resolved infrared nanoimaging and nanospectroscopy of polaritons in a liquid environment. *Nano Letters* **2021**, *21 (3)*, 1360–1367

S25. Ni, G.; McLeod, A.S.; Sun, Z.; Matson, J.R.; Lo, C.F.B.; Rhodes, D.A.; Ruta, F.L.; Moore, S.L.; Vitalone, R.A.; Cusco, R.; Artus, L.; Xiong, L,; Dean, C.R.; Hone, J.C.; Millis, A.J.; Fogler, M.M.; Edgar, J.H.; Caldwell, J.D.; Basov, D.N. Long-lived phonon polaritons in hyperbolic materials. *Nano Letters* **2021**, *21 (13)*, 5767–5773

S26. Chaudhary, K.; Tamagnone, M.; Yin, X.; Spägele, C.M.; Oscurato, S.L.; Li, J.; Persch, C.; Li, R.; Rubin, N.A.; Jauregui, L.A.; Watanabe, K.; Taniguchi, T.; Kim, P.; Wuttig, M.; Edgar, J.H.; Ambrosio, A.; Capasso, F. Polariton nanophotonics using phase-change materials. *Nature Communications* **2019**, *10 (1)*, 4487

S27. Teng, H.; Chen, N.; Hu, H.; García de Abajo, F. J.; Dai, Q. Steering and cloaking of hyperbolic polaritons at deep-subwavelength scales. *Nature Communications* **2024**, *15 (1)*, 4463

S28. Guo, X.; Wu, C.; Zhang, S.; Hu, D.; Zhang, S.; Jiang, Q.; Dai, X.; Duan, Y.; Yang, X.; Sun, Z.; Zhang, S.; Xu, H.; Dai, Q. Mid-infrared analogue polaritonic re-

versed Cherenkov radiation in natural anisotropic crystals. *Nature Communications* **2023**, *14 (1)*, 2532

S29. Zhang, Q.; Ou, Q.; Hu, G.; Liu, J.; Dai, Z.; Fuhrer, M.S.; Bao, Q.; Qiu, C; Hybridized hyperbolic surface phonon polaritons at α-MoO_3 and polar dielectric interfaces. *Nano Letters* **2021**, *21 (7)*, 3112–3119

S30. Gonzalo, Á.P.; Folland, T.G.; Errea, I.; Javier, T.G.; Jiahua, D.; Javier, M.S.; Javier, T.M.; Ana, I.F.T,M.; Matson, J.R.; Bylinkin, A.; Mingze, H.; Ma, W.; Bao, Q.; Martin, J.I.; Caldwell, J.D.; Nikitin, A.Y.; Alonso-GonzÁlez, P. Infrared permittivity of the biaxial van der waals semiconductor α-MoO_3 from near-and far-field correlative studies. *Advanced Materials* **2020**, *32 (29)*, 1908176

S31. Álvarez-Pérez, G.; Voronin, K.V.; Volkov, V.S.; Alonso-González, P.; Nikitin, A.Y. Analytical approximations for the dispersion of electromagnetic modes in slabs of biaxial crystals. *Physical Review B* **2019**, *100 (23)*, 235408

S32. Kresse, G.; Furthmüller, J. Efficient iterative schemes for ab initio total-energy calculations using a plane-wave basis set. *Physical Review B* **1996**, *54 (16)*, 11169

S33. Kresse, G.; Hafner, J.; Ab initio molecular dynamics for liquid metals. *Physical Review B* **1993**, *47 (1)*, 558

S34. Monkhorst, H.J.; Pack, J.D.; Special points for Brillouin-zone integrations. *Physical Review B* **1976**, *13 (12)*, 5188

S35. Langreth, D.C.; Dion, M.; Rydberg, H.; Schröder, E.; and Hyldgaard, P.; and Lundqvist, BI; Van der Waals density functional theory with applications. *International Journal of Quantum Chemistry* **2005**, *101 (5)*, 599-610

S36. Tong, Z.; Dumitrică, T.; Frauenheim, T. First-principles prediction of infrared phonon

and dielectric function in biaxial hyperbolic van der Waals crystal α-MoO_3. *Physical Chemistry Chemical Physics* **2021**, *23 (35)*, 19627–19635

S37. Madou, M.J.; Fundamentals of microfabrication: the science of miniaturization. *CRC press* **2018**,

S38. Tseng, A.A.; Nanofabrication: fundamentals and applications. *World Scientific* **2008**,

S39. Kittel, C.; McEuen, P.; Introduction to solid state physics. *John Wiley & Sons* **2018**,

S40. Adler, R.B.; Smith, A.C.; Longini, R.L.; McKelvey, J.P.; Introduction to semiconductor physics. *American Journal of Physics* **1965**, *33 (11)*, 977–977

S41. Cheung, S.K.; Cheung, N.W., Extraction of Schottky diode parameters from forward current-voltage characteristics. *Applied physics letters* **1986**, *49 (2)*, 85–87

S42. Paparoni, F.; Di Cicco, A.; Minicucci, M.; Mijit, E.; Rezvani, S.J.; A New Cell for In Situ High-Temperature Micro-Raman Experiments: Studying the Dynamics of Oxygen Vacancy Formation in α-MoO_3. *The Journal of Physical Chemistry C* **2024**, *128 (37)*, 15522–15532

S43. Cao, H., Sun, L., Sun, S., Wang, Z., Xie, X., Zhu, T., Zhong, R., Wang, Y., Zhang, K., Mo boosted carrier density of $MoO_{3\text{-x}}$ for surface-enhanced Raman spectroscopy. *Optics Communications* **2025**, *574*, 131066

S44. Julien, C.; Nazri, G.A., Transport properties of lithium-intercalated MoO_3. *Solid State Ionics* **1994**, *68 (1-2)*, 111–116

S45. Maier, S.A., Plasmonics: fundamentals and applications. *Springer* **2007**, *1*,

S46. Spann, B.T.; Compton, R.; Ratchford, D.; Long, J.P.; Dunkelberger, A.D.; Klein, P.B.; Giles, A.J.; Caldwell, J.D.; Owrutsky, J.C., Photoinduced tunability of the reststrahlen band in 4 H-SiC. *Physical Review B* **2016**, *93(8)*, 085205

S47. Dunkelberger, A.D.; Ellis, C.T.; Ratchford, D.C.; Giles, A.J.; Kim, M.; Kim, C.S.; Spann, B.T.; Vurgaftman, I.; Tischler, J.G.; Long, J.P. and Glembocki, O.J.; Active tuning of surface phonon polariton resonances via carrier photoinjection. *Nature Photonics* **2018**, *12(1)*, 50-56

S48. Garcia-Pomar, J.L.; Fandan, R.; Calle, F.; Pedrós, J.; Modulation of surface phonon polaritons in MoO_3 via dynamic doping of SiC substrate. *Nanophotonics* **2025**, *14(1)*, 23-32

S49. Chen, S.; Bylinkin, A.; Wang, Z.; Schnell, M.; Chandan, G.; Li, P.; Nikitin, A.Y.; Law, S.; Hillenbrand, R.; Real-space nanoimaging of THz polaritons in the topological insulator Bi_2Se_3. *Nature communications* **2022**, *13(1)*, 1374

S50. Balendhran, S.; Deng, J.; Ou, J.Z.; Walia, S.; Scott, J.; Tang, J.; Wang, K.L.; Field, M.R.; Russo, S.; Zhuiykov, S.; Strano, M.S.; Enhanced charge carrier mobility in two-dimensional high dielectric molybdenum oxide. *Advanced Materials* **2013**, *25(1)*, 109–114

S51. Scanlon, D.O.; Watson, G.W.; Payne, D.J.; Atkinson, G.R.; Egdell, R.G.; Law, D.S.L.; Theoretical and experimental study of the electronic structures of MoO_3 and MoO_2. *The Journal of Physical Chemistry C* **2010**, *114(10)*, 4636–4645

S52. Zheng, W.; Cao, W.; Wang, Z.; Deng, H.; Shi, J.; Xiong, R.; Improvement of the thermoelectric properties of a MoO_3 monolayer through oxygen vacancies. *Beilstein journal of nanotechnology* **2019**, *10(1)*, 2031–2038

S53. Qasrawi, A.; Abu, S.M.; Khanfar, H.; Haifa, K.; Structural, optical and dielectric performance of molybdenum trioxide thin films sandwiched with indium sheets. *Digest Journal of Nanomaterials and Biostructures* **2020**,

S54. Caughey, D.Mo.; Thomas, R.E.; Carrier mobilities in silicon empirically related to doping and field. *Proceedings of the IEEE* **1967**, *55(12)*, 2192–2193

S55. Negishi, H.; Negishi, S.; Kuroiwa, Y.; Sato, N.; Aoyagi, S.; Anisotropic thermal expansion of layered MoO_3 crystals. *Physical Review B* **2004**, *69 (6)*, 064111

S56. Yates, B.; Overy, M.J.; Pirgon, O.; The anisotropic thermal expansion of boron nitride: I. experimental results and their analysis. *Philosophical Magazine* **1975**, *32(4)*, 847–857

S57. Singh, B., Gupta, M.K., Mishra, S.K., Mittal, R., Sastry, P.U., Rols, S., Chaplot, S.L.; Anomalous lattice behavior of vanadium pentaoxide (V_2O_5): X-ray diffraction, inelastic neutron scattering and ab initio lattice dynamics. *Physical Chemistry Chemical Physics* **2017**, *19(27)*, 17967–1798

S58. Becker, P., Scyfried, P., Siegert, H.; The lattice parameter of highly pure silicon single crystals. *Zeitschrift für Physik B Condensed Matter* **1982**, *48(1)*, 17–21

S59. Souder, W.H., Hidnert, P.; Thermal expansion of insulating materials. *US Government Printing Office* **1919**, *352*,

S60. Polyakova, I.G.; 4. The main silica phases and some of their properties. *Glass* **2014**, *352*, 197–268

S61. Hussain, N.; Ahmed, S.; Tepe, H.U.; Ullah, K.; Shehzad, K.; Wu, H.; Shcherbakov, M.R.; Giant thermomechanical bandgap modulation in quasi-2D tellurium. *Advanced Functional Materials* **2024**, *34 (46)*, 2407812

S62. Hussain, N.; Ahmed, S.; Tepe, H.U.; Huang, K.; Avishan, N.; He, S.; Rafique, M.; Farooq, U.; Kasirga, T.S.; Bek, A.; Turan, R.; Shehzad, K; Wu, H.; Wang, Z; Ultra-narrow linewidth photo-emitters in polymorphic selenium nanoflakes. *Small* **2022**, *18 (52)*, 2204302

S63. Hussain, N.; Yisen, Y.; Sagar, R.U.R.; Anwar, T.; Murtaza, M.; Huang, K.; Shehzad, K.; Wu, H.; Wang, Z.; Quantum-confined blue photoemission in strain-engineered few-atomic-layer 2D germanium. *Nano Energy* **2021**, *83*, 105790

S64. Hu, Y., Liu, X., Xu, S., Wei, W., Zeng, G., Yuan, H., Gao, Q., Guo, J., Chao, M. and Liang, E.; Improving the thermal expansion and capacitance properties of MoO3 by introducing oxygen vacancies. *The Journal of Physical Chemistry C* **2021**, *125(19)*, 10817-10823

S65. Cai, Q., Scullion, D., Gan, W., Falin, A., Zhang, S., Watanabe, K., Taniguchi, T., Chen, Y., Santos, E.J. and Li, L.H.; .High thermal conductivity of high-quality monolayer boron nitride and its thermal expansion. *Science advances* **2019**, *5(6)*, p.eaav0129
